# Digital technology and nursing care: a scoping review on acceptance, effectiveness and efficiency studies of informal and formal care technologies

**DOI:** 10.1186/s12913-019-4238-3

**Published:** 2019-06-20

**Authors:** Tobias Krick, Kai Huter, Dominik Domhoff, Annika Schmidt, Heinz Rothgang, Karin Wolf-Ostermann

**Affiliations:** 1 SOCIUM Research Center on Inequality and Social Policy, University of Bremen, Mary-Somerville-Straße 3, 28359 Bremen, Germany; 2 Institute for Public Health and Nursing Research, University of Bremen, Grazer Straße 4, 28359 Bremen, Germany; 3 High-profile Area of Health Sciences, University of Bremen, Bremen, Germany

**Keywords:** Technology, Care, Nursing, Scoping Review, Efficiency, Effectiveness, Acceptance, Evaluation, Effect, Digital

## Abstract

**Background:**

The existence, usage and benefits of digital technologies in nursing care are relevant topics in the light of the current discussion on technologies as possible solutions to problems such as the shortage of skilled workers and the increasing demand for long-term care. A lack of good empirical overviews of existing technologies in the present literature prompted us to conduct this review. Its purpose was to map the field of digital technologies for informal and formal care that have already been explored in terms of acceptance, effectiveness and efficiency (AEE), and to show the scope of the used methods, target settings, target groups and fields of support.

**Methods:**

A systematic literature search was conducted using Medline, Scopus, CINAHL, Cochrane Library, ACM Digital Library, IEEE Xplore, the Collection of Computer Science Bibliographies, GeroLit and CareLit. In addition, project websites were manually screened for relevant publications.

**Results:**

Seven hundred fifteen papers were included in the review. Effectiveness studies have been most frequently performed for ICT, robots and sensors. Acceptance studies often focussed on ICT, robots and EHR/EMR. Efficiency studies were generally rare. Many studies were found to have a low level of evidence. Experimental designs with small numbers and without control groups were the most common methods used to evaluate acceptance and effectiveness. Study designs with high evidence levels were most commonly found for ICT, robots and e-learning. Technologies evaluated for informal caregivers and children or indicated for formal care at home or in cross-sectoral care were rare.

**Conclusion:**

We recommend producing high-quality evaluations on existing digital technologies for AEE in real-life settings rather than systematic reviews with low-quality studies. More focus should be placed on research into efficiency. Future research should be devoted to a closer examination of the applied AEE evaluation methods. Policymakers should provide funding to enable large-scale, long-term evaluations of technologies in the practice of care, filling the research gaps for technologies, target settings and target groups identified in this review.

**Electronic supplementary material:**

The online version of this article (10.1186/s12913-019-4238-3) contains supplementary material, which is available to authorized users.

## Background

Digital technologies promise great opportunities to overcome existing problems and challenges in the care sector. Many health care systems face challenges such as a shortage of skilled workers and, simultaneously, an increasing demand for long-term care owing to demographic change [1]. Research activities on digital technologies and care are flourishing, nurtured by the expectation that information technologies can help people in need of care to maintain their independence and improve their quality of life and health [2], and also support formal and informal caregivers. Initial studies emphasize positive effects of electronic systems on, for example, patient safety and improvements in the care process [3], which could help to make the best possible use of the available resources.

The German cooperative research project “Pflegeinnovationszentrum” (Nursing Care Innovation Centre), funded by the Federal Ministry of Education and Research (BMBF), aims at establishing a competence centre for innovations in nursing care. Its intention is to collate and produce evidence on the acceptance, effectiveness, and efficiency (AEE) of digital technologies in nursing care and translate these findings into practice. This includes the translation of competencies on these technologies into nursing education A first, essential step of the project is to assess the broad range of technologies developed to support nursing care and nursing education and to provide an overview on existing evidence relating to the AEE of these technologies by conducting this review. We are interested in these outcome dimensions because they can indicate whether a technology has a realistic chance to be transferred into nursing practice. The scope of the existing literature on technology in nursing care and nursing education is very broad. In the present scoping review, we aim to provide insight into the full scope of studies containing information on AEE for informal and formal care.

There is a large number of small-scale studies that explore individual technologies for informal and formal care in the present literature. For example, electronic point-of-care wound documentation for residential long-term care [4], noise-sensor light alarms for the intensive care unit [5], companion robots for elderly care [6] or multi-municipal support networks for informal carers [7]. Virtual reality technology is tested in nursing education [8] and nursing homes use electronic medical records to organize their patient data and thereby optimize their performance [9]. Existing overview articles usually focus on individual technologies [10–14] or on specific target groups like stroke survivors [15], often in combination with single outcome dimensions, such as effectiveness [11], acceptance [16] or efficiency [17]. Still, many systematic reviews in the field of technology and nursing care resume that solid evidence with respect to effectiveness and efficiency of the investigated technologies is still missing or scarce [11, 18–23]. To the best of our knowledge, there is no review article that outlines the broad range of technologies developed to support formal and informal care, and no research findings are available that outline the existing evidence with respect to AEE for this broad field of technologies. This study thus makes a significant contribution to the overview of the entire study scope on the subject of digital technology and nursing care covering all areas of informal and formal care, including nursing education. The study contributes to reveal for which areas of technology there may be evidence that qualifies to be justifiably analysed in detail and for which areas solid research on AEE needs to be intensified.

### Objective and research question

The ultimate objective of this scoping review is to identify technology areas that are promising for further research, to identify current research gaps and to examine how research is conducted [24]. We therefore aim to map the field of digital technologies for informal and formal care that have already been explored in terms of AEE and to show the scope of the used methods, target settings, fields of support and target groups of these technologies. This scoping review should enable researchers to identify the areas of technologies for which it is necessary to systematically analyse the existing evidence and for which areas of technologies further research is necessary. Since our aim is therefore not only to summarize well-researched technologies, but also to identify less-researched technologies that have so far been studied at a low level of evidence, a scoping review is the appropriate method.

This review is thus guided by the following main research questions:

- (i) Which areas of digital technologies aiming to support informal or formal care are most frequently researched with respect to all outcome dimensions (AEE)? (ii) Which target settings, fields of support and target groups are addressed in these studies? (iii) Which study designs have been used to analyse the outcome dimensions?

## Methods

### Methodological basis

Our scoping review was conducted on the basis of Arksey and O’Malley’s scoping review framework [25]. Additional processual advice by Levac, Colquhoun et al. [26] was taken into consideration to enhance the scientific process. The processual advices were particularly used for the identification of relevant studies by balancing comprehensiveness with the feasibility of resources and the iterativity of the team process to select, extract and chart the data.

### Data sources

The database search included the following nine electronic databases: Medline, Scopus, CINAHL, Cochrane Library, ACM Digital Library, IEEE Xplore, the Collection of Computer Science Bibliographies, GeroLit and CareLit. An additional hand-search of relevant projects from German-speaking countries was carried out to supplement the results. The literature search was carried out in March 2018. Due to the large number of studies found, the reference lists of the included studies were not scrutinized.

### Eligibility criteria

We included scientific papers that were published between 2011 and 2018, contained empirical studies (abstract available) in German or English language. All Databases have been searched in March 2018, which limits the included time period from January 2011 to March 2018. The considered time period was limited to 7 years, to make the scope manageable and to focus on the most innovative developments.

Included papers had to report study results relating to acceptance, effectiveness (including efficacy) or efficiency (including cost analysis) of digital technologies in nursing care and nursing education. Such technologies were required to i) either support the immediate action of a caregiver or ii) contribute to the self-reliance of the person in need of care in such a way that direct on-site care assistance can be waived, or iii) substitute the nursing support by using technology or iv) support the training or education of nurses. The assistance of the technology may relate to the person in need of care, formal caregivers, informal caregivers or organizational processes. It potentially involves a wide range of technical innovations. Target settings that have been included are residential long-term care, formal and informal care at home, hospital care, cross-sectoral care, palliative inpatient care, intensive care unit (ICU) care, day-care centre care.

We excluded studies i) without human participation; ii) situated in an emergency department, rehabilitation or surgery context; iii) comprising the following technologies: solely mechanical devices and aids, electrical devices that are not networked or that do not rely on sensors to detect the activity of the person in need of care or caregiver or their immediate vicinity, biotechnology, nanotechnology, medical devices (unless very closely related to nursing activities), imaging diagnostics, tissue engineering, devices with functional diagnostic focus, invasive technologies, mobile visits, telemedicine services, purely pleasure-oriented games, textile technology and technical evaluations of algorithms. Excluded settings and technologies were chosen in alignment with the underlying project.

### Search Terms

The search terms selected were based on an initial literature review and the available knowledge of experts involved in this project. Each term has been adapted to the respective format of each database. German equivalents have been used for the two German databases (GeroLit and CarelLit). All search queries can be provided upon request.

### English search strategy

(Care OR Caring OR Nursing) AND (Technol* OR Robot* OR Intelligent OR Smart OR Assistive OR Decision Support System OR Ambient Assisted Living OR Sensor OR Wearable OR Virtual Reality OR Mixed Reality OR Tagging OR Tracking OR Remote Health Monitoring OR Fall Detection OR Human Computer Interaction OR Human Machine Interaction OR Gerontotechnology OR Gerontechnology OR Head Mounted Display OR Exoskeleton OR Augmented Reality OR Biomedical Monitoring) AND (Effectiveness OR Efficacy OR Effect OR Efficiency OR Acceptance OR Adoption OR Acceptability HTA OR Health Technology Assessment OR Evaluation OR Evaluations OR Cost-Benefit Analysis OR Cost Benefit OR Cost Effectiveness OR Cost Utility OR Cost Analysis OR Cost Analyses OR Cost Consequence OR Economic Evaluation OR Economic Evaluations OR Economic Analysis OR Economic Analyses OR Costs and Benefits OR Benefits and Costs OR Costs and Outcomes OR Marginal Analysis)

### German search strategy

(Pflege) UND (Techn* ODER Technik ODER Robot* ODER Computer ODER Maschine ODER Smart ODER Intelligent ODER Assistive ODER Ambient assisted living ODER Sensor ODER Wearable ODER Virtual reality ODER Mixed reality ODER Ortung ODER Sturzerkennung ODER Mensch-Maschine-Interaktion ODER Gerontechnologie ODER Head mounted display ODER Exoskelett ODER Augmented reality ODER Biomedizinisches Monitoring) UND (Effektivität ODER Effektivität ODER Effizienz ODER Evaluation ODER Akzeptanz ODER Adoption ODER Technikakzeptanz ODER HTA ODER Health technology assessment ODER Kosten ODER Nutzen ODER Kosten-Nutzen-Analyse ODER Wirksamkeit ODER Gesundheitsökonomische Analyse ODER Marginalanalyse)

### Identifying relevant studies

We imported all search results into EndNote X8 and reimported all titles and abstracts into the Excel screening workbook by VonVille [27]. Two researchers independently screened 100 titles and Cohen's kappa was calculated to verify agreement between the reviewers on the inclusion and exclusion criteria. The eligibility criteria were refined until a good agreement of 0.716 was reached. Two pairs of two reviewers each independently screened half of the titles and abstracts. A third person was consulted in case of disagreement on whether an article should be included. The eligibility criteria were then refined again before screening the full texts to reach a maximum consensus on criteria. Considering the large number of full texts to be screened in relation to the existing resources, we created a control scheme whereby each author screened a part of the full texts and, in case of exclusion, a further author checked whether the exclusion criteria matched.

### Data extraction

A data extraction form was collectively drawn up in Excel and piloted to record authors, year, title, abstract, country, study design, number of study participants, technology category, outcome dimension, target setting, field of support of the technology and the addressed target groups. Patterns were filtered out from a digital, automated data analysis [28], as well as from previous interviews with experts and an initial literature search, to develop an optimal technology category system. We iteratively added categories if technologies were found that did not fit into any previously known pattern. Sixteen technology categories were drawn up to classify the technologies discussed in each article. Most of the categories still comprise a wide range of technologies. In a final step, the extraction form was optimized and adapted for all categories in an iterative team process. Four authors screened the full texts and extracted information. Each full text was reviewed once if it was clearly classified with the extraction form. If a text was excluded, a second author checked the reason and re-included if necessary.

### Methodological quality appraisal

In line with guidelines for conducting a scoping review, no formal assessment of methodological quality of the included articles was performed [25, 26, 29].

### Charting the data

During the analysis phase, we iteratively reviewed the results to find an adequate means of presenting the descriptive numerical data. Despite this process we observed that a non-overlapping categorization of individual technologies was not possible due to the complexity of the technologies and their interconnectedness. Since we were aware of this issue from the beginning, we refined the categories in many revision processes to guarantee the best possible classification system. Technologies were assigned to the most fitting category; for instance, although a robot presented in the study has multiple sensors, it is classified as a robot, not a sensor. The importance of all results for both the practical implementation and the study situation were then discussed in a team process [26].

## Results

### Search results

**Fig. 1:**
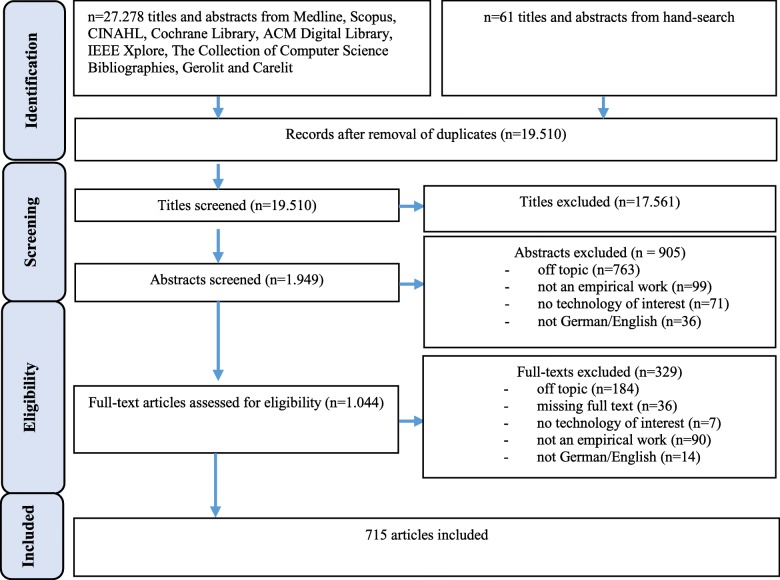
Search results and publication selection process

A total of 27.339 articles were retrieved for this review, including 27.278 from the databases and 61 from hand-search. After removing duplicates, 19.510 remained for screening the titles. 1.949 articles were chosen from screening the abstracts, yielding 1.044 full-texts eligible for full-text screening. 715 full texts were included for the data analysis (see PRISMA flow diagram in Fig. 1). The studies included came from 69 different countries. A complete list of all contained studies can be found in Additional file 1.

### Technology categories

We analysed the number of included studies on each technology area to identify which technology areas were most frequently explored in terms of all outcome dimensions (AEE), and which were least frequently researched. An overview of the distribution of included studies in terms of technology categories is presented in Table 1. The table is sorted by frequencies. A lack of universal definitions for different technology categories, was clearly noticeable during the analysis of the studies. The definitions we developed to differentiate the technologies in this review are included in Table 1. The most widely researched technology category is Information and Communication Technologies (ICT) (*n* = 147). ICT comprises a wide range of technologies. In general, ICT are technologies that provide or document relevant information with a primary focus on improve interpersonal communication. Included technologies can be found in Table 1. Electronic Health Records (EHR)/ Electronic Medical Records (EMR), Hospital/Care Institution Information Systems (HIS) or monitoring technologies could also be included in the category ICT. Since these areas represent large fields of research, we have decided to present them separately. The second most frequently researched category is robots (*n* = 102). We found that the robots under scrutiny here differ greatly in their focus. They provide support on numerous different levels, e.g. physical, psychological, social, organisational, security or educational and therapeutic. All types of robots that were called “robot” in the article are grouped in this category.

**Table 1:** Technology categories with included studies

Category	Definitions	Number of included studies
ICT	ICT are technologies, that either provide or document relevant information, support data management and transfer and focus mostly on improvement of interpersonal communication. The category comprises for example Telecare, Tele-ICU or software applications for process planning.	147
Robot	Robots are machines that interact with their physical environment by sensors, actuators and information technology. This includes social assistive robots, physical assistive robots and complex robotic systems.	102
Sensor	Sensors measure physical or chemical properties and are used to assess, e.g. behaviour, movements or odours. They are often used to control/trigger other devices like pumps or alarm systems.	83
Multiple Technologies	Interventions/studies that include technologies from different technology categories.	80
EHR/EMR	Electronic health records (EHR) and electronic medical records (EMR) are digital records of patient related health information. EMR refers to patient data that is stored and exchanged inside an institution, mostly a hospital. The main focus of the EHR is the capability to exchange information between two systems.	57
Monitoring	Monitoring technologies are complex and analytical technologies to monitor patient, caregiver or organisational relevant data over a period. They often integrate sensors but are more complex than single sensor solutions.	51
Assistive Device	Assistive Devices assist or support a caregiver or a person in need of care in performing a particular task and are enhanced with digital technology, i.e. are digitally connected or equipped with sensor technology.	39
E-Learning	E-Learning includes forms of learning that use electronic or digital media to present or distribute learning resources, or to support communication in learning settings. [30]	38
HIS	Hospital/Care Institution Information Systems (HIS) collect, store, manage and transmit data in hospitals or other care institutions. They can comprise operational management systems, EMR and/or other organisational systems.	30
Educational Technology	Educational Technologies assist learning in nursing education by simulating real life care scenarios and/or incorporate feedback systems. Examples are high fidelity simulations and nurse self-training systems.	23
AAL	AAL technologies are integrated multifunctional, often modular systems that support a person in his/her living environment. AAL generally comprises a set of different technologies, often sensors and communication technologies, that intend to support the well-being, security and independent living of an elderly person. [31]	18
Decision Support	Decision support systems are software solutions that link individual patient data (input) with treatment guidelines and a recommendation (output) to be delivered to a person in charge of care. [12]	18
Virtual Reality	Virtual Reality refers to non-immersive as well as fully immersive, 360-degree artificial environment, which is experienced through a head mounted display (HMD). [32]	11
Tracking	Tracking technologies locate people or objects.	9
Serious Games	Serious Games aim to develop, improve or help maintaining certain skills or competencies, or to evoke behaviour changes.	8
PMR	Personal medical records (PMR) are digital records of patient related health information, that are accessible for patients.	1
Total		715

The third most frequently researched technology category is sensors (*n* = 83). These sensors can either aim at measuring behaviour, movement, falls and other parameters or to measure in combination with controlling other devices like pumps or alarm systems. Many studies cover multiple technologies (*n* = 80) rather than one technology only. Most of them are reviews that focus on specific target groups or nursing problems. A large share of these studies are acceptance studies that comprise a range of different technologies. Only few studies actually provide research on the effectiveness or efficiency of technological systems comprising different types of technologies. Less researched technologies are virtual reality (VR) technologies (*n* = 11) that create a virtual world, tracking technologies (*n* = 9) intended to locate either people or objects, and serious games, which are used for learning purposes or to improve personal independence. We found only one study on personal medical records (PMR), which – in contrast to EMR – allows patients access to all their data. Still, depending on the classification system, PMR could also be subordinated to studies on EMR. This study should therefore not be given a special status. In summary, ICT, robot and sensor technologies can be stated as the most frequently explored areas of technology in terms of all outcome dimensions (AEE). VR, tracking technologies and serious games are the least researched technologies.

### Outcome dimensions and technologies

The inclusion criteria of this study comprise a broad understanding of the outcome dimensions “acceptance”, “effectiveness” and “efficiency”. This is reflected in the broad scope of conceptualizations of these outcome dimensions in the studies included and widely differing measurement concepts. Acceptance studies include the quantitative measurement of acceptance in accordance with a wide range of theoretical acceptance models as well as qualitatively described acceptance results. Effectiveness comprises results on the technical effectiveness or accuracy of technologies as well as personal health or care-related outcomes, organisational or learning outcomes. As there are only very few studies focussing on costs of technologies at all, studies categorized as efficiency-studies include simple cost analyses next to very few full economic evaluations.

With respect to the specific outcome dimensions (AEE), 60 % of all included studies (*n* = 427) analyse aspects of the effectiveness of care technologies, 59 % (*n* = 424) analyse acceptance and only 5,8 % (*n* = 42) analyse efficiency or at least included a cost analysis. Multiple counts of studies are possible, because some studies consider multiple outcome dimensions, which is why the percentage shares add up to more than 100 %. A detailed analysis by outcome dimension (Table 2) shows that acceptance studies are most frequently performed for ICT (*n* = 93), followed by robots (*n* = 64) and EMR/EHR (*n* = 48). Studies on effectiveness have been most frequently carried out for ICT (*n* = 94). Sensor technologies represent the second largest group (*n* = 68) and robotic technologies make up the third (*n* = 57). Efficiency has been studied very rarely for all technologies. ICT (*n* = 9) can be highlighted for this category. Still, compared to the considerably high total number of ICT studies, only 6% of them cover efficiency or cost analyses. In summary, we have found a large number of effectiveness studies with a focus on ICT, robots and sensors, and a large number of acceptance studies focusing on ICT, robots, and EHR/EMR. Efficiency studies are very rare.

**Table 2:** Number of studies by technology category and study outcome dimensions

Technology	Outcome Dimensions			
	Acceptance	Effectiveness	Efficiency	Total number of studies
ICT	93	94	9	147
Robot	64	57	1	102
Sensor	47	68	5	83
Multiple Technologies	25	48	7	80
EHR/EMR	48	17	5	57
Monitoring	26	32	4	51
Assistive Device	25	24	3	39
E-Learning	18	26	0	38
HIS	25	11	3	30
Educational Technology	12	15	0	23
AAL	15	7	2	18
Decision Support	6	13	3	18
Virtual Reality	7	6	0	11
Tracking	6	5	0	9
Serious Games	6	4	0	8
PMR	1	0	0	1
Total	424	427	42	715

### Target settings and technologies

The most frequently researched technologies and their target settings are depicted in Table 3. Most of the included studies aim at hospital care (*n* = 169), which accounts for almost a quarter of all included studies (about 24%). Studies on technologies for informal care at home represent 21% (*n* = 147) and studies on technologies for residential long-term care make up 17% of the studies included (*n* = 122). Ninety-one articles left the setting undefined (13 %). These are more or less explorative studies researching general aspects of the technology in question without considering specific applications. It is noticeable that technologies for formal care (*n* = 48) at home are much less intensively researched than technologies for informal care at home. Studies on technologies for formal care at home account for only 6.7% of all included studies. Hardly any studies focus on cross-sectoral care (<1%).

**Table 3:** Number of studies by technology category and specific target setting

Technology	Target Setting										
	Hospital care	Informal care at home	Residential long-term care	Formal care at home	ICU care	Cross sectoral care	Day-care centre care	Education	Palliative inpatient care	N.A.	Undefined
ICT	42	28	10	22	16	3	3	9	0	3	15
Robot	5	22	46	2	1	0	3	2	0	2	23
Sensor	12	26	16	2	6	0	0	0	1	0	21
Multiple Technologies	15	19	12	7	1	0	0	6	0	9	15
EHR/EMR	33	2	10	1	4	3	0	3	0	0	1
Monitoring	12	23	6	2	3	0	0	0	1	0	5
Assistive Device	12	8	3	6	1	0	0	0	0	0	9
E-Learning	0	0	0	0	0	0	0	38	0	0	0
HIS	22	0	6	1	4	0	0	0	0	0	0
Educational Technology	0	0	0	0	0	0	0	23	0	0	0
AAL	0	11	4	4	0	0	0	0	0	0	0
Decision Support	9	3	3	0	1	0	0	0	0	1	0
Virtual Reality	4	0	2	0	0	0	0	5	0	0	0
Tracking	3	4	1	1	0	0	0	0	0	0	1
Serious Games	0	0	3	0	0	0	0	4	0	0	1
PMR	0	1	0	0	0	0	0	0	0	0	0
Total	169	147	122	48	37	6	6	90	2	15	91

Regarding the most common technologies by setting, ICT (*n* = 42), EHR/EMR (*n* = 33) and HIS (*n* = 22) are most frequently researched in hospital care. The use of medication administration systems [33–35], a multilingual translation aid [36] and the use of a smartphone nurse call system [37] are typical applications for ICT ins this domain. In the informal home-care setting ICT (*n* = 28), sensors (*n* = 26) and monitoring technologies (*n* = 23) are the most commonly used. Sensors, for example, often check activities of everyday life or abnormal behaviours such as falls [38]. In the field of residential long-term care, robots are by far the most researched technology category, followed by sensors (*n* = 16) and ICT (*n* = 10). Social robots [39], therapeutic robots [40] and also robotic auxiliary systems such as robotic transport assistants [41] can be highlighted as common applications. Studies situated in formal care at home mostly focus on ICT *n* = 22). One purpose of ICTs in this setting is communication between nurses and other health professionals, such as general practitioners, in order to obtain sufficient patient-relevant information [42].

We found very few studies on virtual reality (VR) technologies in the literature. Studies on VR were performed only in hospitals (*n* = 4), residential long-term care (*n* = 2) and in the field of education (*n* = 5). Distraction therapy for pain patients in hospital can be cited as an example for the use of this technology [43]. In education, VR is used primarily in terms of VR learning simulations [44–46]. In summary, most of the included technologies are for hospital care, informal care at home and residential long-term care. There is also a large number of studies in which the setting remains undefined. Only a few studies focus on formal care at home, and hardly any on cross-sectoral care.

### Field of support and technologies

We also analysed the fields of support that the technologies are promoting (Table 4). Most technology applications included in this review aim at providing organisational support (*n* = 169). This corresponds to a share of 24% of all included studies. Work organization, self-management and organisational support in everyday life are included in this category. Organisational support is most commonly pursued by ICT (*n* = 49) and EHR/EMR (*n* = 48). Many technologies aim not just at one field, but at multiple areas. Technologies supporting several areas account for 21% of all included studies (*n* = 162). Security-related technologies make up around 14% of all included studies, thus forming another important support area (*n* = 99). Sensors are the most commonly explored security support technology (*n* = 45). Physical (*n* = 46), social (*n* = 40) or psychological support systems are relatively less explored. In the included studies, robotic systems are most frequently employed to provide support in one of these three categories. Technologies that focus mainly on economic support (*n* = 2) are rather uncommon. The total results in 713 studies, because two studies could not be assigned to a field of support. In summary, most of the included studies on technologies aim to provide support at the organisational level (work- and self-organisation) and in the field of security. Furthermore, there is a large number of technologies that aim at multiple support areas. Technologies that provide physical, psychological, social or economic support were explored less often.

**Table 4:** Number of studies by technology category and specific field of support

Technology	Field of support									
	Organisational	Security	Educational	Monitoring	Physical	Psychological	Social	Economic	Multiple	Total
ICT	49	15	12	4	3	8	9	0	45	145
Robot	9	2	3	0	21	13	27	0	27	102
Sensor	2	45	0	21	4	3	1	0	7	83
Multiple Technologies	12	8	6	2	3	3	2	1	43	80
EHR/EMR	48	2	3	0	0	1	0	0	3	57
Monitoring	3	10	0	33	0	0	0	0	5	51
Assistive Device	5	7	0	1	12	4	0	0	10	39
E-Learning	0	0	38	0	0	0	0	0	0	37
HIS	23	3	0	0	0	0	0	0	4	30
Educational Technology	0	0	23	0	0	0	0	0	0	23
AAL	4	1	0	0	1	0	0	0	12	18
Decision Support	9	1	0	0	0	3	1	1	3	18
Virtual Reality	0	0	5	0	2	3	0	0	1	11
Tracking	4	5	0	0	0	0	0	0	0	9
Serious Games	0	0	4	0	0	2	0	0	2	8
PMR	1	0	0	0	0	0	0	0	0	1
Total	169	99	94	61	46	40	40	2	162	713

### Target groups and technologies

The data analysis of the target groups presented in Table 5 shows which target groups are most frequently addressed by the different technologies. In general, the research on most of the technologies included in this review addresses people in need of care (*n* = 382). Formal caregivers (*n* = 326) represent the second largest target group. Technologies for informal caregivers are relatively rarely explored. Only 8% of all included studies focus on informal caregivers (*n* = 57). Also, technologies that address the institutional level are less explored (6% of all studies). Children in need of care are rarely found as a specific target group in the included studies (*n* = 7). The described trends differ for some of the technology categories. EHR/EMR systems usually address formal caregivers (*n* = 40) and AAL systems mostly target at people in need of care (*n* = 17). Sensors (*n* = 70) and monitoring technologies (*n* = 35) are also primarily used to record the parameters of people in need of care. Whereas educational technologies are exclusively intended for the education of formal caregivers (*n* = 23), educational technologies for informal caregivers or people in need of care themselves are not explored so far in terms of AEE. Most studies on technologies for informal caregivers describe ICT systems that provide better information about the caring process or help in ways of communication with professionals or the people in need of care. In summary, most of the included technologies focus on people in need of care and formal caregivers. Technologies with a focus on children and informal caregivers are much less commonly researched.

**Table 5:** Number of studies by technology category and specific target group

Technology	Formal Caregivers	Informal Caregivers	People in Need of Care		Institution	Undefined
			Total	Children		
ICT	81	25	74	4	4	0
Robot	16	5	97	0	1	0
Sensor	17	1	70	0	0	0
Multiple Technologies	30	14	42	0	11	2
EHR/EMR	40	0	1	0	16	0
Monitoring	17	3	35	2	0	1
Assistive Device	14	2	30	1	0	0
E-Learning	37	0	1	0	0	0
HIS	23	0	1	0	8	0
Educational Technology	23	0	0	0	0	0
AAL	5	4	17	0	0	0
Decision Support	12	1	3	0	2	1
Virtual Reality	5	0	6	0	0	0
Tracking	2	2	8	0	2	0
Serious Games	4	0	4	0	0	0
PMR	0	0	1	0	0	0
Total	326	57	382	7	44	4

### Study design and outcome dimensions

The quality and scope of evidence that is generated in the studies on acceptance, effectiveness and efficiency largely depends on the studies’ designs. We refer to common evidence-based nursing and evidence-based medicine guidelines [47, 48] to assess the evidence level of the different study designs. Based on these guidelines, we categorise meta-analysis, systematic reviews (Ia), RCTs (Ib) and quasi-experiments (II) as the highest levels of evidence, evidence from well-designed cohort studies or case-control studies as a medium level of evidence (III) and evidence from single descriptive, qualitative (IVa) or uncontrolled interventional studies (IVb) as a low level of evidence. Table 6 presents the outcome dimensions, differentiated by study design. About 22% of the studies on acceptance (*n* = 96) and 32% of the studies on effectiveness (*n* = 138) included in this review fall into a study design category that we call “experimental no control (n.c.)”. This study design thus makes up most of the studies on both outcome dimensions. In this category studies are included that tested technical performance and accuracy (with respect to effectiveness), analysed acceptance under laboratory conditions or first effects with no control groups (mostly under laboratory conditions as well). The term "experiment" is used here in a technical understanding that differs from the methodological understanding of "experimental studies" in the social sciences. The experimental testing of technologies with user studies to understand acceptance, usability, feasibility, and technical effects in engineering is often done with small groups of people who "test" the technology in controlled environments to get accurate measurements and / or to answer questions about the technology [49–53]. The term "experimental (n.c.)" used in our study describes these user studies and connects them with other studies widely used in the social sciences. This situation occurs because nursing technologies are located in an intermediate region between the social and technical sciences. This study design is also classified as having a low level of evidence (IVb) according to the referred guidelines [47, 48].

**Table 6:** Number of studies by study design and outcome dimension

Design	Outcome Dimension		
	Acceptance	Effectiveness	Efficiency
Experimental n.c.	96	138	3
Mixed Methods	83	55	5
Qualitative	68	13	0
Case Study	53	36	5
Cross-sectional	51	18	3
Systematic Review	24	45	5
Other Types of Review	19	25	5
Quasi-experiment	14	46	5
RCT	8	30	4
Cohort Study	6	8	1
Modelling Study	1	4	5
Meta-analysis	1	8	1
Case-control	0	1	0
Total	424	427	42

Besides these studies, 20% of the research on acceptance is carried out using mixed methods designs (*n* = 83). Qualitative approaches (16%), case study designs (12%) and cross-sectional analyses (12%) also make up a considerable share. Larger, cross-sectional studies have often been performed on technologies already in use such as EMR/EHR. This analysis shows that a majority of the included studies on acceptance were performed at a relatively weak level of evidence design. We have found only a few quasi-experiments (*n* = 14) and RCTs (*n* = 8) that analysed acceptance, but relatively many systematic reviews (*n* = 24). These reviews tended to include all types of study designs (qualitative and quantitative) [54–57], which are medium level of evidence designs.

The research approaches to measuring effectiveness found in this review are different. In addition to the experimental n.c. designs already mentioned, mixed methods designs (*n* = 56) and quasi-experiments (*n* = 47) were frequently used to measure effects. Mixed methods designs thus account for 13% and quasi-experiments for 12% of all studies on effectiveness in this review. It is notable that 45 systematic reviews and 8 meta-analysis were found, but only 30 RCTs. Consequently, for each single technology the number of available RCTs is very small. This is consistent with the fact that a lot of systematic reviews conclude that the study situation is not sufficient to report meaningful results on effectiveness [11, 18–23], because there are very few high-quality studies.

Efficiency studies are generally rare. Therefore, a common type of study cannot be named. We found efficiency analyses in modelling studies, quasi-experiments, case studies, mixed methods studies, systematic reviews and other types of reviews (each *n* = 5). Most of the studies categorized as efficiency studies contained only cost analyses (*n* = 21). Cost-effectiveness analyses were performed in 13 studies. Studies providing cost-benefit (*n* = 3) or cost-utility analyses (*n* = 1) were even less common. In summary, most of the included studies on acceptance and effectiveness have an experimental n.c. design. In addition, next to mixed method approaches, acceptance was frequently measured qualitatively and effectiveness with quasi-experiments. Efficiency studies have very rarely been carried out and often focus on cost analysis only.

### Study design and technology

It remains to be clarified for which technology categories included in this review studies with a high level of evidence can be found and for which areas such studies can hardly be found. Table 7 lists common study designs of the included articles in relation to the technology categories for which they were applied. We defined meta-analysis, systematic reviews (Ia), RCTs (Ib) and quasi-experiments (II) as having the highest levels of evidence. Nevertheless, it should be kept in mind that the systematic reviews and meta-analyses included in this review not only consist of data from RCTs, and that they often conclude that the quality of included studies was not sufficient. Other study designs may contribute to a greater insight, depending on the outcome dimensions and the research question. We only consider the formal levels of evidence here. Most high level of evidence studies of the described three levels (Ia,Ib,II) can be found for the category ICT (*n* = 33), followed by robots (*n* = 18), e-learning (*n* = 16), sensors (*n* = 10) and assistive devices (*n* = 10).

**Table 7:** Number of studies by technology category and selected study design

Technology				Design					
	Experimental n.c.	Case Study	Qualitative	Cross-sectional	Mixed Methods	Quasi-experiment	RCT	Systematic Review	Meta-analysis
ICT	26	21	18	10	24	15	6	12	0
Robot	43	9	9	1	20	6	9	3	0
Sensor	59	5	2	1	4	5	4	1	0
Multiple Technologies	7	5	9	9	8	0	3	21	0
EHR/EMR	4	9	11	13	10	0	0	2	1
Monitoring	28	5	4	5	4	0	1	1	1
Assistive Device	12	5	5	4	2	2	4	2	2
E-Learning	5	3	3	3	5	9	2	4	1
HIS	2	7	5	4	6	1	1	4	0
Educational Technology	4	3	2	4	3	3	0	1	1
AAL	4	2	0	3	4	1	1	3	0
Decision Support	1	3	1	0	3	2	1	3	2
Virtual Reality	5	1	1	0	0	3	1	0	0
Tracking	4	0	0	2	2	1	0	0	0
Serious Games	3	1	1	0	0	2	1	0	0
PMR	0	0	0	1	0	0	0	0	0
Total	207	79	71	60	95	50	34	56	8

Few studies with a high level of evidence were found for VR (1 RCT), tracking (1 quasi-experiment) and there were no high-evidence studies for PMR. Despite the wide range of technology categories included, only a few of them have been explored comprehensively using methods with a high level of evidence. Since the technology categories include very different individual technologies, a differentiated analysis would be required here to identify particularly good and less well-researched individual technologies. The robot “Paro” can be highlighted as an example for an individual technology, for which we found a total of seven RCT studies [58–64].

To summarize, studies with a higher level of evidence design (meta-analysis, systematic reviews, RCTs, quasi-experiments) were most commonly found for ICT, robots and e-learning. Only a few studies with a high level of evidence have been found for most of the other areas of technology, like for example VR and tracking.

## Discussion

The aim of this study was to map the field of digital technologies for informal and formal care that have already been explored in terms of AEE and to give a structured overview of the used methods, target settings, fields of support and target groups of these technologies. To our knowledge this is the first study trying to provide a quantitative overview over the entire study scope on the subject of digital technology and nursing care, covering all areas of informal and formal care, including nursing education.

ICT, robot and sensor technologies can be stated to be the most frequently explored areas of technology in terms of all outcome dimensions (AEE). Virtual reality technologies, tracking technologies and serious games are technology categories that are comparatively less researched so far. It can be assumed that the most frequently researched technologies have been the most important areas for researchers and research funders in recent years. Without knowing more about the results of the studies, it is reasonable to conclude that this research interest has either been motivated by high expectations for these technologies in terms of supporting nursing care from the perspective of care research and nursing science – or that nursing contexts are application areas of high interest from the perspective of technical sciences developing these technologies. The rarely researched technologies may be promising fields of research for the future.

Taking a closer look at the outcome dimensions, it has been shown that there is a large number of effectiveness studies with a focus on ICT, robots and sensors, and a large number of acceptance studies focusing on ICT, robots and EHR/EMR. However, a large proportion of these studies has a low level of evidence, as will be elaborated below. Efficiency studies are very rare in general. This points to the low consideration of the relationship between benefits and costs of a technology, so far. There could be several reasons for this. One possible reason is that there are not enough high-quality studies that allow a comparison of the effects of a technology with costs in the form of a health economic evaluation. Many technologies are still under development or have never reached the implementation phase. Subsequently, they may not have reached the stage for high-quality studies in real-life settings, which makes it difficult to accurately estimate future costs. Another reason could be that the future costs of a technology are difficult to predict if the technology is currently still in the development phase, since it can be assumed that the future price of a technology will be significantly lower than the current one.

When analysing the target settings of all included articles, we found that most technologies aim at hospital care, informal care at home or residential long-term care. There is also a large proportion of technologies for which the setting remains undefined. We do not consider it expedient to leave the target setting undefined during the development phase of a technology, because it hinders a purposeful development of the technology. Research gaps related to target settings were found for formal care at home and cross-sectoral care. If this is reflected in relation to the target groups for which the technologies were developed, the analysis shows that most of the included technologies focus on people in need of care and formal caregivers. This means that technologies relating to informal care at home are primarily intended for people in need of care and not for informal caregivers. We assume that this is mainly due to the fact that these technologies are often developed to strengthen the independence of people in need of care and hence to prevent the intervention of informal caregivers. Still, this review identifies informal caregivers as an under-represented group in the exploration of digital technologies. Research on technologies for assisting children in need of care has also very seldom been carried out.

We also analysed the fields of support the included technologies are intended for. Most of the technologies included provide support at the organisational level (work and self-organisation) and the field of security. Furthermore, there is a large part of technologies that provide support in multiple areas. This category often refers to research settings, where multiple technologies are combined, such as AAL, or to systematic reviews that focus on a specific target group or setting and thus include several aspects of support. Another large part are ICTs, which combine organizational as well as psychological or social support. Technologies that exclusively support physical, psychological, social or economic areas were relatively rare.

Our analysis also includes a valuation of the study designs used to evaluate AEE. Although there are many studies on different technologies overall, there are only a few studies with a high level of evidence, considering all outcome dimensions. There are significantly more RCTs, quasi-experiments and systematic reviews for effectiveness than for acceptance. Efficiency studies have been carried out very rarely and often focus on cost-analysis only. Given the low number of studies with a high level of evidence, there are only a few studies that can deliver high-evidence results.

Most of the included studies on acceptance and effectiveness were carried out in an experimental n.c. design. This type of study is essential during the development of a technology in order to establish its effectiveness from a technical–scientific point of view. From the perspective of health and nursing science, however, the evidence level is low with respect to the measurement of health or nursing related effects when applied in actual nursing practice. In addition, next to mixed method approaches, acceptance was frequently measured qualitatively and effectiveness using quasi-experiments.

Studies with a higher level of evidence design (meta-analysis, systematic reviews, RCTs, quasi-experiments) were most commonly found for ICT, robots and e-learning. It is important to distinguish between systematic reviews and meta-analyses on the one hand and RCTs and quasi-experiments on the other. A systematic review has a high level of evidence if it is based on studies with high evidence levels. If a systematic review is based on low-quality studies with a low level of evidence, it adds only a little insight into the effectiveness or acceptance of a technology. Therefore, a closer analysis of the systematic reviews and meta-analyses included here in terms of quality and results is needed to finally judge their quality. This applies especially to the area of multiple technologies. However, for ICT, robot and e-learning there are relatively many RCTs and quasi-experiments, so it can be expected that systematic reviews on a high level of evidence are possible in these research areas. Still, a first look at the systematic reviews included in this article reveals that many of them actually conclude that there are not enough high-evidence studies, and more high-quality studies are needed. This also seems to apply to sub-areas of ICT [10], robots [11] e-learning [65], AAL [23] and assistive technologies[19, 20].

Overall, the methods used in all studies appear to be very diverse, and the measured outcome parameters diverge broadly for the different technology categories, which also could be a problem for the subsequent comparability of results of the studies in terms of AEE.

### Limitations

Although our scoping review was conducted in line with the standards of the methodology [25, 26], we still need to acknowledge some limitations. We have ventured into a field with a huge scope. Given the broad field and large number of potentially relevant technologies, producing a concise capture, systematization and summary of all information was indeed a challenging. To make the scope manageable, the considered time period was limited to 7 years. This must be named as a limitation, because no longer period could be displayed. A systematization of all technological innovations without any overlaps was not possible due to the complexity of the technologies. The highest possible quality standards for classification were developed in an iterative team process, but possible overlaps should be taken into account when interpreting the presented results.

Looking at all 715 studies included in this review it was noticeable that a lot of studies describe their methods and results poorly. This made it difficult to evaluate and describe relevant information. The quality of the description of the study tended to increase with the quality of the study design. Still the impression arose that not uncommonly, study authors tended to enhance their study design by labelling it a study design of a higher evidence level than was actually used.

We also had to make methodical compromises due to the available resources, as recommended by Levac et al. [26], but we were still able to maintain the quality by applying the four-eyes principle in all steps of exclusion using a special sequencing method we developed for this review. The publication bias must be mentioned as a further limitation of this review. We considered published scientific studies only, and no grey literature. This review therefore tends to contain fewer publications with negative or neutral findings [66]. Consequently, it can be assumed that there may be a bias towards promising technologies.

There may also be an over-representation of some technology areas, as we have included both systematic reviews and primary studies. Some primary studies are included in the systematic reviews. However, we have accepted this limitation in order to get an overview of the different levels of evidence used to explore individual technology areas.

We did not scrutinize the reference lists of all studies found in the databases, moreover, due to the huge amount of potential publications found at this stage. We are therefore unable to consider technologies in early stages of development and without any published studies involving actual users. Nevertheless, a comprehensive overview of the scope of relevant literature has been provided by our thorough search through nine databases, covering the key areas of health and nursing science as well as the field of computer science.

## Conclusion

The results of this scoping review can be used as a basis for further research in the field of digital technology and nursing care. We mapped the field of technologies for informal and formal care that has already been explored in terms of AEE, and presented a structured overview of the methods used, target settings, fields of support and target groups of these technologies and provide data-based indications which technologies appear to be promising for further research. Given the broad field and large number of potentially relevant technologies, producing a concise capture, systematization and summary of all information was indeed a challenging research.

We recommend that for the time being the scientific community should not focus on conducting systematic reviews on digital technologies in nursing care, because there appears to be a lack of high-quality studies. Rather, we recommend producing high-quality evaluations on existing technologies in terms of acceptance, effectiveness and efficiency in real-life settings. A special focus should be placed on research into efficiency, as – at the time of writing – the proportion of efficiency studies is particularly low. Future research should also be devoted to taking a closer look at the applied evaluation methods for AEE and deciding whether they are appropriate or whether new methods are needed to perform an ideal measurement of AEE. When analysing the target settings and target groups, we found that formal care at home and cross-sectoral care technologies are underexplored in terms of AEE. There are also numerous technologies where the setting remains undefined. We recommend defining an application setting when developing technologies for care. Technologies for informal caregivers and children in need of care have seldom been explored. Policymakers should provide funding to enable large-scale, long-term evaluations of digital technologies in the practice of care, filling research gaps for technologies, target settings and target groups we identified.

## Additional file

Additional file 1: Overview of all included studies. (PDF 572 kb)

## Data Availability

The datasets used and/or analysed during the current study are available from the corresponding author on reasonable request.

## Abbreviations

AAL: Ambient Assisted Living
ACM: Association for Computing Machinery
AEE: Acceptance, Effectiveness and Efficiency
EHR: Electronic Health Records
EMR: Electronic Medical Records
HIS: Hospital/Care Institution Information Systems
HMD: Head Mounted Display
ICT: Information and Communication Technology
ICU: Intensive Care Unit
IEEE: Institute of Electrical and Electronics Engineers Living
PMR: Personal Medical Records
