# Supplementary material for: Digital technology and nursing care: a scoping review on acceptance, effectiveness and efficiency studies of informal and formal care technologies
Source: BMC Health Serv Res. 2019 Jun 20;19:400. doi: 10.1186/s12913-019-4238-3 (PMC6585079; doi:10.1186/s12913-019-4238-3)
Supplement: Supplementary file 1 — Overview of all included studies. (PDF 572 kb) [file 12913_2019_4238_MOESM1_ESM.pdf]

Nr.	Authors	Year	Title
1	M. Aanesen, A. T. Lotherington and F. Olsen	2011	Smarter elder care? A cost-effectiveness analysis of implementing technology in elder care
2	K. S. Abate	2013	The Effect of Podcast Lectures on Nursing Students' Knowledge Retention and Application
3	B. Abdelbari, D. Lieve, M. Eric, a. Frank Van Fraeyenhove and S. Dirk	2012	Falls prevention in a palliative care unit: evaluation of an infrared sensor device
4	A. P. Achilleos, C. Mettouris, G. A. Papadopoulos, K. Neureiter, C. Rappold, C. Moser, M. Tscheligi, L. Vajda, A. Tóth, P. Hanák, O. Jimenez and R. Smit	2013	The connected vitality system: Enhancing social presence for older adults
5	J. Adler-Milstein and A. K. Jha	2017	HITECH Act Drove Large Gains In Hospital Electronic Health Record Adoption
6	S. Ahmadzada, M. A. Zayyad and M. Toycan	2016	Readiness assessment for the use of cloud computing in eHealth systems: A field study of hospitals in the capital of Azerbaijan
7	H. S. Ahn, M. H. Lee, E. Broadbent and B. A. MacDonald	2017	Gathering Healthcare Service Robot Requirements from Young People’s Perceptions of an Older Care Robot
8	C. Aileen Wai-Kiu, C. Sek-Ying, J. Wing-Hung Sit, E. Mi-Ling Wong, D. Tze-Fun Lee and O. Wai-Man Fung	2016	Case-Based Web Learning Versus Face-to-Face Learning: A Mixed-Method Study on University Nursing Students
9	M. Akiyama and Y. Sasaki	2013	Efficacy of the drug administration support system for improving drug compliance in home-care
10	N. Al Saleem and A. Al Harthy	2015	Innovative information systems in the intensive care unit, king saud medical city in Saudi Arabia
11	A. Alaiad and L. Zhou	2015	Patients' Behavioral Intentions toward Using WSN Based Smart Home Healthcare Systems: An Empirical Investigation
12	A. Alaiad and L. Zhou	2017	Patients' Adoption of WSN-Based Smart Home Healthcare Systems: An Integrated Model of Facilitators and Barriers
13	I. Alakärppä, S. Larsson, J. Riekki and E. Jaakkola	2011	Sound aided interface of a pervasive pain monitoring system
14	M. B. Alazzam, A. S. H. Basari, A. S. Sibghatullah, M. R. Ramli, M. M. Jaber and M. H. Naim	2016	Pilot study of EHRs acceptance in Jordan hospitals by UTAUT2
15	U. V. Albrecht, M. Behrends, H. K. Matthies and U. Von Jan	2013	Usage of multilingual mobile translation applications in clinical settings
16	J. Alcalá, O. Parson and A. Rogers	2015	Detecting anomalies in activities of daily living of elderly residents via energy disaggregation and Cox processes

17	A. Y. Aldehaim, F. F. Alotaibi, C. R. Uphold and S. Dang	2016	The Impact of Technology-Based Interventions on Informal Caregivers of Stroke Survivors: A Systematic Review
18	G. L. Alexander, R. W. Madsen, E. L. Miller, M. K. Schaumberg, A. E. Holm, R. L. Alexander, K. K. Wise, M. L. Dougherty and B. Gugerty	2017	A national report of nursing home information technology: year 1 results
19	G. L. Alexander, R. W. Madsen, E. L. Miller, D. S. Wakefield, K. K. Wise and R. L. Alexander	2017	The State of Nursing Home Information Technology Sophistication in Rural and Nonrural US Markets
20	G. L. Alexander, K. S. Pasupathy, L. M. Steege, E. B. Strecker and K. M. Carley	2014	Multi-disciplinary communication networks for skin risk assessment in nursing homes with high IT sophistication
21	G. L. Alexander, L. M. Steege, K. S. Pasupathy and K. Wise	2015	Case studies of IT sophistication in nursing homes: A mixed method approach to examine communication strategies about pressure ulcer prevention practices
22	A. Almutairi and R. M. Crindle	2015	A pilot study in Jeddah City of nurses perceptions of Electronic Medical Records
23	H. Aloulou, M. Mokhtari, T. Tiberghien, J. Biswas, C. Phua, J. H. Kenneth Lin and P. Yap	2013	Deployment of assistive living technology in a nursing home environment: Methods and lessons learned
24	M. H. Alsulami and A. S. Atkins	2016	Factors Influencing Ageing Population for Adopting Ambient Assisted Living Technologies in the Kingdom of Saudi Arabia
25	M. M. Altuwaijri	2011	Achieving excellence in Electronic Health Record deployment in Middle East hospitals
26	A. G. Alvarez and G. T. M. Dal Sasso	2011	Virtual Learning Object for the Simulated Evaluation of Acute Pain in Nursing Students
27	J. Alwin, J. Persson and B. Krevers	2013	Perception and significance of an assistive technology intervention - the perspectives of relatives of persons with dementia*
28	A. Aman	2013	Clinical information systems in private hospitals
29	M. Amiribesheli and A. Bouchachia	2016	Towards Dementia-Friendly Smart Homes
30	T. Ando, M. Takeda, T. Maruyama, Y. Susuki, T. Hirose, S. Fujioka, O. Mizuno, K. Yamada, Y. Ohno and H. Yukio	2013	Biosignal-based relaxation evaluation of head-care robot
31	S. Andreadis, T. G. Stavropoulos, G. Meditskos and I. Kompatsiaris	2016	Dem@home: Ambient intelligence for clinical support of people living with dementia
32	C. M. Angst, S. Devaraj and J. D'Arcy	2012	Dual role of IT-assisted communication in patient care: A validated structure-process-outcome framework
33	A. Appari, E. K. Carian, M. E. Johnson and D. L. Anthony	2012	Medication administration quality and health information technology: a national study of US hospitals

34	A. Appari, E. M. Johnson and D. L. Anthony	2014	Information technology and hospital patient safety: a cross-sectional study of US acute care hospitals
35	C. Arditi, M. Rège-Walther, J. C. Wyatt, P. Durieux and B. Burnand	2012	Computer-generated reminders delivered on paper to healthcare professionals; effects on professional practice and health care outcomes
36	L. E. Arenas, P. J. Bedoya, L. Correa, J. G. Barreneche and A. M. Hernández	2017	Usability evaluation for a vital signs monitor prototype
37	N. Armstrong	2012	Design, development and evaluation of assistive technologies to assist people with Alzheimers disease by supporting their caregivers
38	I. Asghar, S. Cang and H. Yu	2018	Usability evaluation of assistive technologies through qualitative research focusing on people with mild dementia
39	C. C. V. Avelino, F. R. Borges, C. M. Inagaki, M. De Abreu Nery and S. L. T. Goyatá	2016	Development of a course in the virtual learning environment on the ICNP
40	M. Azarm-Daigle, C. Kuziemsky and L. Peyton	2015	A review of cross organizational healthcare data sharing
41	O. Aziz and S. N. Robinovitch	2011	An Analysis of the Accuracy of Wearable Sensors for Classifying the Causes of Falls in Humans
42	D. ÃztÃ¼rk and L. DinÃ§	2014	Effect of web-based education on nursing students' urinary catheterization knowledge and skills
43	M. M. Baig and H. GholamHosseini	2013	Wireless remote patient monitoring in older adults
44	M. M. Baig, H. GholamHosseini, M. J. Connolly and G. Kashfi	2014	A wireless patient monitoring system for hospitalized older adults: Acceptability, reliability and accuracy evaluation
45	S. Baisch, T. Kolling, S. Rühl, B. Klein, J. Pantel, F. Oswald and M. Knopf	2018	Emotional robots in a nursing context: Empirical analysis of the present use and the effects of Paro and Pleo
46	H. U. Balaguera, D. Wise, C. Y. Ng, H. W. Tso, W. L. Chiang, A. M. Hutchinson, T. Galvin, L. Hilborne, C. Hoffman, C. C. Huang and C. J. Wang	2017	Using a Medical Intranet of Things System to Prevent Bed Falls in an Acute Care Hospital: A Pilot Study
47	W. Bani-issa, N. Al Yateem, I. K. Al Makhzoomy and A. Ibrahim	2016	Satisfaction of health-care providers with electronic health records and perceived barriers to its implementation in the United Arab Emirates
48	A. Bankole, M. Anderson, A. Knight, K. Oh, T. Smith-Jackson, M. A. Hanson, A. T. Barth and J. Lach	2011	Continuous, Non-invasive Assessment of Agitation in Dementia Using Inertial Body Sensors

49	A. Barriga, J. M. Conejero, J. Hernández, E. Jurado, E. Moguel and F. Sánchez-Figueroa	2016	A vision-based approach for building telecare and telerehabilitation services
50	M. Baslyman, R. Rezaee, D. Amyot, A. Mouttham, R. Chreyh, G. Geiger, A. Stewart and S. Sader	2015	Real-time and Location-based Hand Hygiene Monitoring and Notification: Proof-of-concept System and Experimentation
51	L. Battista and G. Summa	2016	Preliminary evaluation of a wireless remote monitoring system for home mechanical ventilation
52	U. Bayen, A. Dogangün, T. Grundgeiger, A. Haese, G. Stockmanns and J. Ziegler	2013	Evaluating the effectiveness of a memory aid system
53	E. A. Beccaluva, A. Bonarini, R. Cerabolini, F. Clasadonte, F. Garzotto, M. Gelsomini, V. A. Iannelli, F. Monaco and L. Viola	2017	Exploring engagement with robots among persons with neurodevelopmental disorders
54	K. Beedholm, K. Frederiksen and K. Lomborg	2016	What Was (Also) at Stake When a Robot Bathtub Was Implemented in a Danish Elder Center: A Constructivist Secondary Qualitative Analysis
55	A. N. Belbachir, M. Litzenberger, S. Schraml, M. Hofstätter, D. Bauer, P. Schön, M. Humenberger, C. Sulzbachner, T. Lunden and M. Merne	2012	CARE: A dynamic stereo vision sensor system for fall detection

56	M. Belshaw, B. Taati, J. Snoek and A. Mihailidis	2012	Towards a Single Sensor Passive Solution for Automated Fall Detection?Falling in the home is one of the major challenges to independent living among older adults. The associated costs, coupled with a rapidly growing elderly population, are placing a burden on healthcare systems worldwide that will swiftly become unbearable. To facilitate expeditious emergency care, we have developed an artificially intelligent camera-based system that automatically detects if a person within the field-of-view has fallen. The system addresses concerns raised in earlier work and the requirements of a widely deployable in-home solution. The presented prototype utilizes a consumer-grade camera modified with a wide-angle lens. Machine learning techniques applied to carefully engineered features allow the system to classify falls at high accuracy while maintaining invariance to lighting, environment and the presence of multiple moving objects. This paper describes the system, outlines the algorithms used and presents empirical validation of its effectiveness. I.
57	J. A. G. Beltran, M. C. Leon, A. I. G. Martınez and J. I. N. Hipolito	2014	Health emergency event notification system, towards to the seamless service mobility
58	R. Bemelmans, G. J. Gelderblom, P. Jonker and L. de Witte	2012	Socially Assistive Robots in Elderly Care: A Systematic Review into Effects and Effectiveness
59	R. Bemelmans, G. J. Gelderblom, P. Jonker and L. de Witte	2015	Effectiveness of Robot Paro in Intramural Psychogeriatric Care: A Multicenter Quasi-Experimental Study
60	C. C. Bennett, S. Sabanovic, J. A. Piatt, S. Nagata, L. Eldridge and N. Randall	2017	A Robot a Day Keeps the Blues Away
61	K. Bennett, F. Grasso, V. Lowers, A. McKay and C. Milligan	2015	Evaluation of an App to Support Older Adults with Wounds
62	R. Berenbaum, Y. Lange and L. Abramowitz	2011	Augmentative Alternative Communication for Alzheimer?s Patients and Families? Using SAVION
63	S. D. Bersch, C. M. J. Chislett, D. Azzi, R. Kusainov and J. S. Briggs	2011	Activity detection using frequency analysis and off-the-shelf devices: Fall detection from accelerometer data
64	M. Bettinelli, Y. Lei, M. Beane, C. Mackey and T. Liesching	2015	Does Robotic Telerounding Enhance Nurse-Physician Collaboration Satisfaction About Care Decisions?

65	L. M. Beuscher, F. Jing, N. Sarkar, M. S. Dietrich, P. A. Newhouse, K. F. Miller and L. C. Mion	2017	Socially Assistive Robots: Measuring Older Adults' Perceptions
66	K. C. Bezboruah, D. Paulson and J. Smith	2014	Management attitudes and technology adoption in long-term care facilities
67	P. Bhattarai and J. Phillips	2017	The role of digital health technologies in management of pain in older people: an integrative review
68	A. A. Bindakheel, A. Bulgiba and R. Omar	2014	Adoption of information communication technology at a hospital: A case study of the King Fahad Medical City
69	M. Birks, M. Bodak, J. Barlas, J. Harwood and M. Pether	2016	Robotic Seals as Therapeutic Tools in an Aged Care Facility: A Qualitative Study
70	M. Birks, P. Hartin, C. Woods, E. Emmanuel and M. Hitchins	2016	Students' perceptions of the use of eportfolios in nursing and midwifery education
71	J. D. Blakey, D. Guy, C. Simpson, A. Fearn, S. Cannaby, P. Wilson and D. Shaw	2012	Multimodal observational assessment of quality and productivity benefits from the implementation of wireless technology for out of hours working
72	D. Blum, S. X. Raj, R. Oberholzer, I. I. Riphagen, F. Strasser and S. Kaasa	2015	Computer-Based Clinical Decision Support Systems and Patient-Reported Outcomes: A Systematic Review
73	M. E. Bobillier Chaumon, S. Bekkadja, F. Cros and B. Cuvillier	2014	The user-centered design of an ambient technology for preventing falls at home
74	W. Boonchieng, E. Boonchieng, W. Tuanrat, C. Khuntichot and K. Duangchaemkarn	2017	Integrative system of virtual electronic health record with online community-based health determinant data for home care service: MHealth development and usability test
75	R. G. Booth, B. Sinclair, G. Strudwick, L. Brennan, J. Tong, H. Relouw, M. Hancock and W. Vlasic	2017	Identifying Error Types Made by Nursing Students Using eMAR Technology
76	E. Børøsund, C. M. Ruland, S. Moore and M. Ekstedt	2014	Nurses' experiences of using an interactive tailored patient assessment tool one year past implementation
77	S. Bouakaz, M. Vacher, M. E. Bobillier Chaumon, F. Aman, S. Bekkadja, F. Portet, E. Guillou, S. Rossato, E. Desserée, P. Traineau, J. P. Vimont and T. Chevalier	2014	CIRDO: Smart companion for helping elderly to live at home for longer
78	D. J. Bouvier, J. G. Hinz and C. A. Schmidt	2016	Pilot Study: User Acceptance of a Virtual Coach in a Mirror by Elderly Persons with Dementia

79	A. Bowes, A. Dawson and Greasley-Adams	2013	Literature review: the cost effectiveness of assistive technology in supporting people with dementia. Report to the Demtia Services Development Trust
80	K. H. Bowles, A. L. Hanlon, H. A. Glick, M. D. Naylor, M. O'Connor, B. Riegel, N. W. Shih and M. G. Weiner	2011	Clinical effectiveness, access to, and satisfaction with care using a telehomecare substitution intervention: a randomized controlled trial
81	L. Bowtell, A. A. Kist, D. Osbourne and V. Parker	2013	Improving clinical practice outcomes for nurses with an interactive emulator
82	L. Bowtell, C. Moloney, A. A. Kist, V. Parker, A. Maxwell and N. Reedy	2012	Using Remote Access Laboratories in nursing education
83	A. Boyd, J. Synnott, C. Nugent, D. Elliott and J. Kelly	2017	Community-based trials of mobile solutions for the detection and management of cognitive decline
84	S. L. Bradley	2011	A phenomenological exploration of nurses' perceptions of the effect of electronic documentation on healing relationships
85	D. Bragg, N. Huynh and R. E. Ladner	2016	A Personalizable Mobile Sound Detector App Design for Deaf and Hard-of-Hearing Users
86	Å. Brandt, J. Alwin, H. Anttila, K. Samuelsson and A.-L. Salminen	2012	Quality of evidence of assistive technology interventions for people with disability: An overview of systematic reviews
87	F. Braun, A. Lemkaddem, V. Moser, S. Dasen, O. Grossenbacher and M. Bertschi	2017	Contactless respiration monitoring in real-time via a video camera
88	C. Bräutigam, P. Enste, M. Evans, J. Hilbert, J. Merkel and F. Öz	2017	Digitalisierung im Krankenhaus. Mehr Technik - bessere Arbeit?
89	T. J. Bright, A. Wong, R. Dhurjati, E. Bristow, L. Bastian, R. R. Coeytaux, G. Samsa, V. Hasselblad, J. W. Williams, M. D. Musty, L. Wing, A. S. Kendrick, G. D. Sanders and D. Lobach	2012	Effect of clinical decision-support systems: A systematic review
90	E. Broadbent, N. Kerse, K. Peri, H. Robinson, C. Jayawardena, T. Kuo, C. Datta, R. Stafford, H. Butler, P. Jawalkar, M. Amor, B. Robins and B. MacDonald	2016	Benefits and problems of health-care robots in aged care settings: A comparison trial
91	E. Broadbent, J. R. Orejana, H. S. Ahn, J. Xie, P. Rouse and B. A. MacDonald	2015	The cost-effectiveness of a robot measuring vital signs in a rural medical practice
92	M. Browning, S. Cooper, R. Cant, L. Sparkes, F. Bogossian, B. Williams, P. O'Meara, L. Ross, G. Munro and B. Black	2016	The use and limits of eye-tracking in high-fidelity clinical scenarios: A pilot study

93	J. R. Bruun-Pedersen, S. Serafin and L. B. Kofoed	2016	Going Outside While Staying Inside — Exercise Motivation with Immersive vs. Non–immersive Recreational Virtual Environment Augmentation for Older Adult Nursing Home Residents
94	B. B. Bundschuh, R. W. Majeed, T. Bürkle, K. Kuhn, U. Sax, C. Seggewies, C. Vosseler and R. Röhrig	2011	Quality of human-computer interaction - Results of a national usability survey of hospital-IT in Germany
95	M. B. Buntin, M. F. Burke, M. C. Hoaglin and D. Blumenthal	2011	The Benefits Of Health Information Technology: A Review Of The Recent Literature Shows Predominantly Positive Results
96	A. Bygholm and A. M. Kanstrup	2014	Learning from an Ambient Assisted Living Lab: The case of the Intelligent Bed
97	R. G. Cady	2012	Measuring the Impact of Technology on Nurse Workflow: A Mixed Methods Approach
98	R. G. Cady and S. M. Finkelstein	2014	Task-technology fit of video telehealth for nurses in an outpatient clinic setting
99	H. Cai, E. Toft, O. Hejlesen, J. Hansen, C. Oestergaard and B. Dinesen	2015	Health professionals' user experience of the intelligent bed in patients' homes
100	D. Calvaresi, D. Cesarini, P. Sernani, M. Marinoni, A. F. Dragoni and A. Sturm	2017	Exploring the ambient assisted living domain: a systematic review
101	W. Cao, X. Liu and F. Li	2017	Robust device-free fall detection using fine-grained Wi-Fi signatures
102	D. Capurro, M. Ganzinger, J. Perez-Lu and P. Knaup	2014	Effectiveness of eHealth interventions and information needs in palliative care: a systematic literature review
103	P. Carayon, R. Cartmill, M. A. Blosky, R. Brown, M. Hackenberg, P. Hoonakker, A. S. Hundt, E. Norfolk, T. B. Wetterneck, J. M. Walker, P. Carayon, R. Cartmill, M. A. Blosky, R. Brown, M. Hackenberg, P. Hoonakker, A. S. Hundt, E. Norfolk, T. B. Wetterneck and J. M. Walker	2011	ICU nurses' acceptance of electronic health records
104	K. J. Carlson and D. J. Gagnon	2016	Augmented Reality Integrated Simulation Education in Health Care
105	S. H. Carpenter	2016	What deters nurses from participating in web-based graduate nursing programs?: A cross-sectional survey research study

106	S. Carretero, J. Stewart and C. Centeno	2015	Information and communication technologies for informal carers and paid assistants: benefits from micro-, meso-, and macro-levels
107	V. Castilho, A. F. C. Lima, F. M. T. Fugulin, H. H. C. Peres and R. R. Gaidzinski	2014	Total staff costs to implement a decision support system in nursing
108	L. Cattelani, F. Chesani, P. Palumbo, L. Palmerini, S. Bandinelli, C. Becker and L. Chiari	2014	FRAT-Up, a Rule-Based System Evaluating Fall Risk in the Elderly
109	F. Cavallo, M. Aquilano, M. Bonaccorsi, R. Limosani, A. Manzi, M. C. Carrozza and P. Dario	2013	On the design, development and experimentation of the ASTRO assistive robot integrated in smart environments
110	J. G. Cegarra-Navarro, A. K. P. Wensley and M. T. SÃ¡nchez-Polo	2011	Improving quality of service of home healthcare units with health information technologies
111	M. Chang, T. Yu, J. Luo, K. Duan, P. Tu, Y. Zhao, N. Nagraj, V. Rajiv, M. Priebe and M. Stachura	2017	Multi-Modal Sensor System for Pressure Ulcer Wound Assessment and Care
112	Y. J. Chang, C. H. Chen, L. F. Lin, R. P. Han, W. T. Huang and G. C. Lee	2012	Wireless sensor networks for vital signs monitoring: Application in a nursing home
113	S. Changping	2011	Application of SimMan universal patient simulator in the teaching of medical nursing
114	P. Chanyagorn, B. Kungwannarongkun and W. Chanyagorn	2016	Design of electronic nursing Kardex system for medication error prevention in IPD patients
115	Y. Charlon, E. Campo and D. Brulin	2018	Design and evaluation of a smart insole: Application for continuous monitoring of frail people at home
116	Y. Charlon, N. Fourty, W. Bourennane and E. Campo	2013	Design and evaluation of a device worn for fall detection and localization: Application for the continuous monitoring of risks incurred by dependents in an Alzheimer's care unit
117	N. Charness	2014	Utilizing Technology to Improve Older Adult Health
118	P. Chaurasia, S. I. McClean, C. D. Nugent, I. Cleland, S. Zhang, M. P. Donnelly, B. W. Scotney, C. Sanders, K. Smith, M. C. Norton and J. Tschanz	2016	Modelling assistive technology adoption for people with dementia
119	C. Chen, D. Zhang, L. Sun, M. Hariz and B. Jean-Bart	2013	AQUEDUC: improving quality and efficiency of care for elders in real homes
120	S. T. Chen, Y. G. L. Huang and I. T. Chiang	2012	Using Somatosensory Video Games to Promote Quality of Life for the Elderly with Disabilities

121	A. Cheng, W. Eppich, V. Grant, J. Sherbino, B. Zendejas and D. A. Cook	2014	Debriefing for technology-enhanced simulation: a systematic review and meta-analysis
122	Y. M. Cheng	2013	Exploring the roles of interaction and flow in explaining nurses' e-learning acceptance
123	J. Cherian, V. Rajanna, D. Goldberg and T. Hammond	2017	Did You Remember to Brush?: A Noninvasive Wearable Approach to Recognizing Brushing Teeth for Elderly Care
124	N. Chi and G. Demiris	2015	A systematic review of telehealth tools and interventions to support family caregivers
125	K. F. Chiang and H. H. Wang	2016	Nurses' experiences of using a smart mobile device application to assist home care for patients with chronic disease: A qualitative study
126	J. M. Ching, B. L. Williams, L. M. Idemoto and C. C. Blackmore	2014	Using Lean 'Automation with a Human Touch' to Improve Medication Safety: A Step Closer to the 'Perfect Dose'
127	C. Chi-Ping, L. Ting-Ting, L. Chia-Hui and M. E. Mills	2016	Nurses' Experiences of an Initial and Reimplemented Electronic Health Record Use
128	J. Chipps, P. Brysiewicz and M. Mars	2012	A Systematic Review of the Effectiveness of Videoconference-Based Tele-Education for Medical and Nursing Education
129	Y.-S. Choi, E. Lawler, C. A. Boenecke, E. R. Ponatoski and C. M. Zimring	2011	Developing a multi-systemic fall prevention model, incorporating the physical environment, the care process and technology: a systematic review
130	S. K. Y. Chow, W.-Y. Chin, H.-Y. Lee, H.-C. Leung and F.-H. Tang	2012	Nurses' perceptions and attitudes towards computerisation in a private hospital
131	S. Christiansen and A. Rethmeier	2015	Preparing Student Nurses for the Future of Wound Management: Telemedicine in a Simulated Learning Environment
132	M.-T. Chu, R. Khosla, S. M. S. Khaksar and K. Nguyen	2017	Service innovation through social robot engagement to improve dementia care quality
133	S. T. Chuang, Y. F. Liu, Z. X. Fu, K. C. Liu, S. H. Chien, C. L. Lin and P. Y. Lin	2015	Application of a smartphone nurse call system for nursing care
134	J. Chung and I. Cho	2017	The need for academic electronic health record systems in nurse education
135	J. Chung, G. Demiris, H. J. Thompson, K.-Y. Chen, R. Burr, S. Patel and J. Fogarty	2017	Feasibility testing of a home-based sensor system to monitor mobility and daily activities in Korean American older adults
136	J. Chung, H. J. Thompson, J. Joe, A. Hall and G. Demiris	2017	Examining Korean and Korean American older adults' perceived acceptability of home-based monitoring technologies in the context of culture
137	N. J. Cibulka and L. Crane-Wider	2011	Introducing Personal Digital Assistants to Enhance Nursing Education in Undergraduate and Graduate Nursing Programs

138	V. Claes, E. Devriendt, J. Tournoy and K. Milisen	2015	Attitudes and perceptions of adults of 60 years and older towards in-home monitoring of the activities of daily living with contactless sensors: An explorative study
139	J. Clark and M. McGee-Lennon	2011	A stakeholder-centred exploration of the current barriers to the uptake of home care technology in the UK
140	C. N. Clarke, S. H. Patel, R. W. Day, S. George, C. Sweeney, G. A. Monetes De Oca, M. A. Aiss, E. G. Grubbs, B. K. Bednarski, J. E. Lee, D. C. Bodurka, J. M. Skibber and T. A. Aloia	2017	Implementation of a standardized electronic tool improves compliance, accuracy, and efficiency of trainee-to-trainee patient care handoffs after complex general surgical oncology procedures
141	C. Cohen, T. Kampel and H. Verloo	2016	Acceptability of an intelligent wireless sensor system for the rapid detection of health issues: findings among home-dwelling older adults and their informal caregivers
142	M. Colombo, E. Marelli, R. Vaccaro, E. Valle, S. Colombani, E. Polesel, S. Garolfi, S. Fossi and A. Guaita	2012	Virtual reality for persons with dementia: An exergaming experience
143	E. J. Cook, G. Randhawa, A. Guppy, C. Sharp, G. Barton, A. Bateman and J. Crawford-White	2017	Exploring factors that impact the decision to use assistive telecare: perspectives of family care-givers of older people in the United Kingdom
144	F. Corno, L. D. Russis and A. M. Roffarello	2016	A Healthcare Support System for Assisted Living Facilities: An IoT Solution
145	H. Courtney-Pratt, E. Cummings, P. Turner, H. Cameron-Tucker, R. Wood-Baker, E. Walters and A. Robinson	2012	Entering a world of uncertainty: community nurses' engagement with information and communication technology
146	K. Cresswell, A. Majeed, D. W. Bates and A. Sheikh	2012	Computerised decision support systems for healthcare professionals: an interpretative review
147	Y. Dahl and K. Holbø	2012	Value Biases of Sensor-based Assistive Technology: Case Study of a GPS Tracking System Used in Dementia Care
148	F. DallaLibera, Y. Tsusaka, Y. Okazaki, R. Futakuchi, M. Yamamoto, N. Shikata and M. Terashima	2016	Analysis of velocity's influence on forces and muscular activity in the context of sit-to-stand motion assisted by an elderly care robot
149	J. Damant, M. Knapp, P. Freddolino and D. Lombard	2017	Effects of digital engagement on the quality of life of older people
150	A. Davies, L. Rixon and S. Newman	2013	Systematic review of the effects of telecare provided for a person with social care needs on outcomes for their informal carers
151	S. R. W. de Almeida, G. T. M. Dal Sasso and D. C. C. Barra	2016	Computerized nursing process in the Intensive Care Unit: Ergonomics and usability

152	J. C. De Gagne	2011	The impact of clickers in nursing education: A review of literature
153	F. S. N. de Góes, L. M. M. Fonseca, R. A. A. de Camargo, G. F. de Oliveira and H. R. Felipe	2015	Educational technology "Anatomy and Vital Signs": Evaluation study of content, appearance and usability
154	E. Delbreil and G. Zvobgo	2013	Wireless sensor technology in dementia care : Caregiver perceptions, technology take-up and business model innovation
155	C. M. DesRoches, P. Miralles, P. Buerhaus, R. Hess and K. Donelan	2011	Health information technology in the workplace: Findings from a 2010 national survey of registered nurses
156	A. Di Nuovo, F. Broz, N. Wang, T. Belpaeme, A. Cangelosi, R. Jones, R. Esposito, F. Cavallo and P. Dario	2017	The multi-modal interface of Robot-Era multi-robot services tailored for the elderly
157	A. Di Nuovo, N. Wang, F. Broz, T. Belpaeme, R. Jones and A. Cangelosi	2016	Experimental evaluation of a multi-modal user interface for a robotic service
158	N. Dimitrioglou, D. Kardaras and S. Barbounaki	2017	Multicriteria Evaluation of the Internet of Things Potential in Health Care: The Case of Dementia Care
159	M. Ding, R. Ikeura, Y. Mori, T. Mukai and S. Hosoe	2013	Measurement of human body stiffness for lifting-up motion generation using nursing-care assistant robot — RIBA
160	M. Ding, R. Ikeura, Y. Mori, T. Mukai and S. Hosoe	2014	Lift-up motion generation of nursing-care assistant robot based on human muscle force and body softness estimation
161	M. Ding, R. Ikeura, T. Mukai, H. Nagashima, S. Hirano, K. Matsuo, M. Sun, C. Jiang and S. Hosoe	2012	Comfort estimation during lift-up using nursing-care robot — RIBA
162	G. D'Onofrio, O. James, D. Sancarlo, F. Ricciardi, K. Murphy, F. Giuliani, D. Casey and A. Greco	2016	Managing active and healthy aging with use of caring service robots (MARIO)
163	G. D'Onofrio, O. James, D. Sancarlo, F. Ricciardi, K. Murphy, F. Giuliani, D. Casey and A. Greco	2016	Evaluation of the acceptability of a caring service robot (MARIO)
164	G. D'Onofrio, D. Sancarlo, F. Ricciardi, F. Panza, D. Seripa, F. Cavallo, F. Giuliani and A. Greco	2017	Information and Communication Technologies for the Activities of Daily Living in Older Patients with Dementia: A Systematic Review
165	C. N. Doukas and I. Maglogiannis	2011	Emergency Fall Incidents Detection in Assisted Living Environments Utilizing Motion, Sound, and Visual Perceptual Components
166	F. Drews and A. Doig	2014	Evaluation of a configural vital signs display for intensive care unit nurses

167	I. Dubovi, S. T. Levy and E. Dagan	2017	Now I know how! The learning process of medication administration among nursing students with non-immersive desktop virtual reality simulation
168	J. J. Duvall	2012	Motivation and technological readiness in the use of high-fidelity simulation: A descriptive comparative study of nurse educators
169	J. A. dx.Nielsen and L. Mathiassen	2013	Interpretive flexibility in mobile health: Lessons from a government-sponsored home care program
170	P. C. Dykes, E. H. I-Ching, J. R. Soukup, F. Chang and S. Lipsitz	2012	A case control study to improve accuracy of an electronic fall prevention toolkit
171	A. D. Edgcomb	2014	Automated Video-Based Fall Detection
172	M. Eldib, F. Deboeverie, D. V. Haerenborgh, W. Philips and H. Aghajan	2015	Detection of Visitors in Elderly Care Using a Low-resolution Visual Sensor Network
173	M. Eldib, F. Deboeverie, W. Philips and H. Aghajan	2016	Behavior analysis for elderly care using a network of low-resolution visual sensors
174	A. Ennis, J. Rafferty, J. Synnott, I. Cleland, C. Nugent, A. Selby, S. McIlroy, A. Berthelot and G. Masci	2017	A smart cabinet and voice assistant to support independence in older adults
175	B. Erol, M. G. Amin and B. Boashash	2017	Range-Doppler radar sensor fusion for fall detection
176	M. Z. Eslami, A. Zarghami, M. v. Sinderen and R. Wieringa	2013	Care-giver tailoring of IT-based healthcare services for elderly at home: A field test and its results
177	A. M. M. C. Espingardeiro	2014	A roboethics framework for the development and introduction of social assistive robots in elderly care
178	I. Eyers, B. Carey-Smith, N. Evans and R. Orpwood	2013	Safe and sound? Night-time checking in care homes
179	U. Fachinger, B. Schöpke and S. Helten	2015	Zur ökonomischen Relevanz von Lösungen zur Sturzerkennung
180	M. Fagan, C. Kilmon and V. Pandey	2012	Exploring the adoption of a virtual reality simulation: The role of perceived ease of use, perceived usefulness and personal innovativeness
181	C. Fagerström, H. Tuvesson, L. Axelsson and L. Nilsson	2017	The role of ICT in nursing practice: an integrative literature review of the Swedish context
182	J. Fan, D. Bian, Z. Zheng, L. Beuscher, P. A. Newhouse, L. C. Mion and N. Sarkar	2017	A Robotic Coach Architecture for Elder Care (ROCARE) Based on Multi-User Engagement Models
183	K. Fan, P. Wang and S. Zhuang	2018	Human fall detection using slow feature analysis

184	B. A. Farshchian and Y. Dahl	2015	The Role of ICT in Addressing the Challenges of Age-related Falls: A Research Agenda Based on a Systematic Mapping of the Literature
185	A. Febretti, K. D. Lopez, J. Stifter, A. E. Johnson, G. Keenan and D. Wilkie	2014	Evaluating a Clinical Decision Support Interface for End-of-life Nurse Care
186	M. Ferrari, B. Harrison, O. Rawashdeh, R. Hammond, Y. Avery, M. Rawashdeh, W. Sa'deh and M. Maddens	2012	Clinical Feasibility Trial of a Motion Detection System for Fall Prevention in Hospitalized Older Adult Patients
187	R. Fiedler, J. Giddens and S. North	2014	Faculty Experience of a Technological Innovation in Nursing Education
188	S. H. Fischer, D. David, B. H. Crotty, M. Dierks and C. Safran	2014	Acceptance and use of health information technology by community-dwelling elders
189	R. Fleming and S. Sum	2014	Empirical studies on the effectiveness of assistive technology in the care of people with dementia: A systematic review
190	B. Florczak, A. Scheurich, J. Croghan, P. Sheridan Jr, D. Kurtz, W. McGill and B. McClain	2012	An observational study to assess an electronic point-of-care wound documentation and reporting system regarding user satisfaction and potential for improved care
191	M. Fossum, M. Ehnfors, A. Fruhling and A. Ehrenberg	2011	An evaluation of the usability of a computerized decision support system for nursing homes
192	K. Funate, R. Tasaki, T. Miyoshi, K. Kakihara and K. Terashima	2017	Motion control of novel power assist lift robot integrated with omnidirectional assist vehicle considering suppression of limit cycle at grounding
193	M. Gams, E. Dovgan, B. Cvetković, V. Mirćevska, B. Kaluža, M. Luštrek and I. Velez	2011	AAL for supporting elderly
194	J. P. García-Vázquez, M. D. Rodríguez, Á. G. Andrade and J. Bravo	2011	Supporting the Strategies to Improve Elders? Medication Compliance by Providing Ambient Aids
195	J. E. Garrido, V. M. R. Penichet, M. D. Lozano and J. A. F. Valls	2013	Automatic detection of falls and fainting
196	C. Garripoli, M. Mercuri, P. Karsmakers, P. J. Soh, G. Crupi, G. A. E. Vandenbosch, C. Pace, P. Leroux and D. Schreurs	2015	Embedded DSP-based telehealth radar system for remote in-door fall detection
197	M. Ghorbel, S. Betgé-Brezetz, M. P. Dupont, G. B. Kamga, S. Piekarec, J. Reerink and A. Vergnol	2013	Multimodal notification framework for elderly and professional in a smart nursing home
198	G. Gibson, C. Dickinson, K. Brittain and L. Robinson	2015	The everyday use of assistive technology by people with dementia and their family carers: A qualitative study

199	D. Gillham, K. Tucker, S. Parker, V. Wright and C. Kargillis	2015	CaseWorld™: Interactive, media rich, multidisciplinary case based learning
200	C. Göransson, I. Eriksson, K. Ziegert, Y. Wengström, A. Langius-Eklöf, M. Brovall, A. Kihlgren and K. Blomberg	2017	Testing an app for reporting health concerns-Experiences from older people and home care nurses
201	M. Gövercin, S. Meyer, M. Schellenbach, E. Steinhagen-Thiessen, B. Weiss, M. Haesner and M. Gövercin	2016	SmartSenior@home: Acceptance of an integrated ambient assisted living system. Results of a clinical field trial in 35 households
202	J. L. Grady	2011	The Virtual Clinical Practicum: An Innovative Telehealth Model for Clinical Nursing Education
203	T. Greenhalgh, J. Wherton, P. Sugarhood, S. Hinder, R. Procter and R. Stones	2013	What matters to older people with assisted living needs? A phenomenological analysis of the use and non-use of telehealth and telecare
204	P. M. Grice, M. D. Killpack, A. Jain, S. Vaish, J. Hawke and C. C. Kemp	2013	Whole-arm tactile sensing for beneficial and acceptable contact during robotic assistance
205	H. M. Gross, S. Mueller, C. Schroeter, M. Volkhardt, A. Scheidig, K. Debes, K. Richter and N. Doering	2015	Robot companion for domestic health assistance: Implementation, test and case study under everyday conditions in private apartments
206	H. M. Gross, C. Schroeter, S. Mueller, M. Volkhardt, E. Einhorn, A. Bley, T. Langner, M. Merten, C. Huijnen, H. v. d. Heuvel and A. v. Berlo	2012	Further progress towards a home robot companion for people with mild cognitive impairment
207	A. Grunerbl, G. Bahle, P. Lukowicz and F. Hanser	2011	Using Indoor Location to Assess the State of Dementia Patients: Results and Experience Report from a Long Term, Real World Study
208	P. Guitard, H. Sveistrup, A. Fahim and C. Leonard	2013	Smart grab bars: a potential initiative to encourage bath grab bar use in community dwelling older adults
209	C. Gustafsson, C. Svanberg and M. Müllersdorf	2015	Using a Robotic Cat in Dementia Care
210	R. M. Hall	2013	Effects of High Fidelity Simulation on Knowledge Acquisition, Self-Confidence, and Satisfaction with Baccalaureate Nursing Students Using the Solomon-Four Research Design
211	V. Hall, S. Conboy-Hill and D. Taylor	2011	Using virtual reality to provide health care information to people with intellectual disabilities: Acceptability, usability, and potential utility
212	M. Hanheide, D. Hebesberger and T. Krajnik	2017	The when, where, and how: an adaptive robotic info-terminal for care home residents ? a long-term study

213	C. Y. N. Hara, N. D. A. Aredes, L. M. M. Fonseca, R. C. d. C. P. Silveira, R. A. A. Camargo and F. S. N. de Goes	2016	Clinical case in digital technology for nursing students' learning: An integrative review
214	Hardin, Sr., J. Dienemann, P. Rudisill and K. Mills	2013	Inpatient fall prevention: use of in-room Webcams
215	C. S. Harmon, M. Fogle and L. Roussel	2015	Then and now: Nurses' perceptions of the electronic health record
216	A. Harris, H. True, Z. Hu, J. Cho, N. Fell and M. Sartipi	2016	Fall recognition using wearable technologies and machine learning algorithms
217	B. J. J. Hattink, F. J. M. Meiland, T. Overmars-Marx, M. de Boer, P. W. G. Ebben, M. van Blanken, S. Verhaeghe, I. Stalpers-Croeze, A. Jedlitschka, S. E. Flick, J. v/d Leeuw, I. Karkowski and R. M. Dröes	2016	The electronic, personalizable Rosetta system for dementia care: exploring the user-friendliness, usefulness and impact
218	H. Hawley-Hague, E. Boulton, A. Hall, K. Pfeiffer and C. Todd	2014	Older adults' perceptions of technologies aimed at falls prevention, detection or monitoring: A systematic review
219	L. Hayden, S. Glynn, T. Hahn, F. Randall and E. Randolph	2012	The use of Internet technology for psychoeducation and support with dementia caregivers
220	K. Heidarizadeh, M. Rassouli, H. Manoochehri, M. Zagheri Tafreshi and R. Kashef Ghorbanpour	2017	Nurses' Perception of Challenges in the Use of an Electronic Nursing Documentation System
221	J. Helmy and A. Helmy	2016	The Alzimio App for Dementia, Autism & Alzheimer's: Using Novel Activity Recognition Algorithms and Geofencing
222	C. Henderson, M. Knapp, J. Fernández, J. Beecham, S. Hirani, M. Beynon, M. Cartwright, L. Rixon, H. Doll, P. Bower, A. Steventon, A. Rogers, R. Fitzpatrick, J. Barlow, M. Bardsley and S. Newman	2014	Cost-effectiveness of telecare for people with social care needs: the Whole Systems Demonstrator cluster randomised trial
223	V. M. Herbert and H. Connors	2016	Integrating an Academic Electronic Health Record: Challenges and Success Strategies
224	B. L. Hicken, C. Daniel, M. Luptak, M. Grant, S. Kilian and R. W. Rupper	2017	Supporting Caregivers of Rural Veterans Electronically (SCORE)
225	V. Hielscher	2014	Technikeinsatz und Arbeit in der_Altenpflege. Ergebnisse einer internationalen Literaturrecherche
226	V. Hielscher, L. Nock and S. Kirchen-Peters	2015	Technikeinsatz in der Altenpflege: Potenziale und Probleme in empirischer Perspektive
227	L. M. Hitt and P. Tambe	2016	Health care information technology, work organization, and nursing home performance

228	R. J. Holden, O. Asan, E. M. Wozniak, K. E. Flynn and M. C. Scanlon	2016	Nurses' perceptions, acceptance, and use of a novel in-room pediatric ICU technology: testing an expanded technology acceptance model
229	B. Holtz and S. Krein	2011	Understanding Nurse Perceptions of a Newly Implemented Electronic Medical Record System
230	E. Hoque, R. F. Dickerson, S. M. Preum, M. Hanson, A. Barth and J. A. Stankovic	2015	Holmes: A Comprehensive Anomaly Detection System for Daily In-home Activities
231	E. T. Horta, I. C. Lopes, J. J. P. C. Rodrigues and M. L. Proença	2013	A mobile health application for falls detection and biofeedback monitoring
232	M. A. Hossain and D. T. Ahmed	2012	Virtual Caregiver: An Ambient-Aware Elderly Monitoring System
233	Y. Hou, N. Li and Z. Huang	2012	Triaxial accelerometer-based real time fall event detection
234	T. V. How, R. H. Wang and A. Mihailidis	2013	Evaluation of an intelligent wheelchair system for older adults with cognitive impairments
235	J. Hsiao, H. Chang and R. Chen	2011	A study of factors affecting acceptance of hospital information systems: a nursing perspective
236	J.-L. Hsiao and R.-F. Chen	2012	An investigation on task-technology fit of mobile nursing information systems for nursing performance
237	S. H. Hsu, J. S. Sun, Y. J. Chou and C. W. Weng	2014	Developing intelligent human-machine interface for next generation ICU by using user-centered system development approach
238	Y. C. Hsu, C. H. Tsai, Y. M. Kuo, Lien and B. Ya-Hui	2016	Telecare services for elderly: Predictive factors of continued use intention
239	C. Hu, S. Kung, T. A. Rummans, M. M. Clark and M. I. Lapid	2015	Reducing caregiver stress with internet-based interventions: a systematic review of open-label and randomized controlled trials
240	N. Hu, R. Bormann, T. Zwölfer and B. Kröse	2014	Multi-user identification and efficient user approaching by fusing robot and ambient sensors
241	H. Huang and T.-t. Lee	2011	Evaluation of ICU nurses' use of the clinical information system in Taiwan
242	H.-Y. Huang and T.-T. Lee	2011	Impact of bar-code medication administration on nursing activity patterns and usage experience in Taiwan
243	Z. Huang, C. Lin, M. Kanai-Pak, J. Maeda, Y. Kitajima, M. Nakamura, N. Kuwahara, T. Ogata and J. Ota	2017	Impact of Using a Robot Patient for Nursing Skill Training in Patient Transfer
244	Z. Huang, A. Nagata, M. Kanai-Pak, J. Maeda, Y. Kitajima, M. Nakamura, K. Aida, N. Kuwahara, T. Ogata and J. Ota	2012	Development of a nursing self-training system for transferring patient from bed to wheelchair

245	Z. Huang, A. Nagata, M. Kanai-Pak, J. Maeda, Y. Kitajima, M. Nakamura, K. Aida, N. Kuwahara, T. Ogata and J. Ota	2014	Self-help training system for nursing students to learn patient transfer skills
246	H. Hung-Hsiou and W. Ya-Hui	2017	Investigation of the Effects of a Nursing Information System by Using the Technology Acceptance Model
247	G. B. Huq, J. Basilakis and A. Maeder	2016	Evaluation of Tri-axial Accelerometery Data of Falls for Elderly Through Smart Phone
248	I. Iacono and P. Marti	2016	Narratives and emotions in seniors affected by dementia: A comparative study using a robot and a toy
249	R. Igual, C. Medrano and I. Plaza	2013	Challenges, issues and trends in fall detection systems
250	P. Irwin and R. Coutts	2015	A Systematic Review of the Experience of Using Second Life in the Education of Undergraduate Nurses
251	C. Ishii, H. Yamamoto and D. Takigawa	2015	Development of a New Type of Lightweight Power Assist Suit for Transfer Work
252	T. Jacobs and B. Graf	2012	Practical evaluation of service robots for support and routine tasks in an elderly care facility
253	R. Janols and B. Göransson	2011	Same System?different Experiences: Physicians? and Nurses? Experiences in Using IT Systems
254	A. d. S. Jayatilaka	2017	Towards technologies for promoting nutritional health in older people with dementia living in their own home
255	C. Jayawardena, I. Kuo, C. Datta, R. Q. Stafford, E. Broadbent and B. A. MacDonald	2012	Design, implementation and field tests of a socially assistive robot for the elderly: HealthBot version 2
256	L. C. Jensen, K. Fischer, S. D. Suvei and L. Bodenhagen	2017	Timing of multimodal robot behaviors during human-robot collaboration
257	L. Jing and Z. Cheng	2017	Recognition of daily routines and accidental event with multipoint wearable inertial sensing for seniors home care
258	H. M. Johnsen, M. Fossum, P. Vivekananda-Schmidt, A. Fruhling and Å. Slettebø	2016	Teaching clinical reasoning and decision-making skills to nursing students: Design, development, and usability evaluation of a serious game
259	K. Johnson and P. Meskill	2012	Online Assessments - What do Students Prefer?
260	P. Jokinen and I. Mikkonen	2013	Teachers' experiences of teaching in a blended learning environment
261	N. Jøranson, I. Pedersen, A. M. M. Rokstad and C. Ihlebæk	2015	Effects on Symptoms of Agitation and Depression in Persons With Dementia Participating in Robot-Assisted Activity: A Cluster-Randomized Controlled Trial

262	N. Jøranson, I. Pedersen, A. M. M. Rokstad and C. Ihlebæk	2016	Change in quality of life in older people with dementia participating in Paro-activity: a cluster-randomized controlled trial
263	C. Jousselme, R. Vialet, E. Jouve, P. Lagier, C. Martin and F. Michel	2011	Efficacy and mode of action of a noise-sensor light alarm to decrease noise in the pediatric intensive care unit: a prospective, randomized study
264	R. Kachouie, S. Sedighadeli, R. Khosla and M. T. Chu	2014	Socially Assistive Robots in Elderly Care: A Mixed-Method Systematic Literature Review
265	E. Kańtoch	2015	BAN-based health telemonitoring system for in-home care
266	C. Karlsen, M. S. Ludvigsen, C. E. Moe, K. Haraldstad and E. Thygesen	2017	Experiences of community-dwelling older adults with the use of telecare in home care services: a qualitative systematic review
267	Y. Kashimoto, T. Morita, M. Fujimoto, Y. Arakawa, H. Suwa and K. Yasumoto	2017	Sensing Activities and Locations of Senior Citizens toward Automatic Daycare Report Generation
268	S. M. S. Khaksar, R. Khosla, M. T. Chu and F. S. Shahmehr	2016	Service Innovation Using Social Robot to Reduce Social Vulnerability among Older People in Residential Care Facilities
269	P. C. B. Khong, S. Y. Hoi, E. Holroyd and W. Wang	2015	Nurses' clinical decision making on adopting a wound clinical decision support system
270	R. Khosla and M. T. Chu	2013	Embodying care in matilda: An affective communication robot for emotional wellbeing of older people in Australian residential care facilities
271	R. Khosla, M. T. Chu, R. Kachouie, K. Yamada and T. Yamaguchi	2012	Embodying care in Matilda - An affective communication robot for the elderly in Australia
272	R. Khosla, M.-T. Chu, R. Kachouie, K. Yamada, F. Yoshihiro and T. Yamaguchi	2012	Interactive Multimodal Social Robot for Improving Quality of Care of Elderly in Australian Nursing Homes
273	P. Khosravi and A. H. Ghapanchi	2016	Investigating the effectiveness of technologies applied to assist seniors: A systematic literature review
274	A. Khunlertkit and P. Carayon	2013	Contributions of tele-intensive care unit (Tele-ICU) technology to quality of care and patient safety
275	B. Kikhia, T. G. Stavropoulos, S. Andreadis, N. Karvonen, I. Kompatsiaris, S. Sävenstedt, M. Pijl and C. Melander	2016	Utilizing a wristband sensor to measure the stress level for people with dementia
276	D. H. Kim, B. MacDonald, A. McDaid, S. Kawamura, H. Kim, E. T. Bean, F. Fraser and E. Broadbent	2016	User perceptions of soft robot arms and fingers for healthcare

277	S. Kim, K. H. Lee, H. Hwang and S. Yoo	2016	Analysis of the factors influencing healthcare professionals' adoption of mobile electronic medical record (EMR) using the unified theory of acceptance and use of technology (UTAUT) in a tertiary hospital
278	B. Kipping, S. Rodger, K. Miller and R. M. Kimble	2012	Virtual reality for acute pain reduction in adolescents undergoing burn wound care: a prospective randomized controlled trial
279	B. Klein and I. Schlömer	2018	A robotic shower system: Acceptance and ethical issues
280	E. Kobayashi, T. Yoshimi, N. Matsuhira, M. Mizukawa and Y. Ando	2015	A study of driving trajectory for standing-up motion support system
281	H. Kobayashi, Y. Harada and K. Tokoro	2014	Development of an independent support system capable of walking from recumbent position
282	T. Kobayashi, K. Katsuragi, T. Miyazaki and K. Arai	2017	SNS Agency Robot for Elderly People Using External Cloud-Based Services
283	Y. Kobayashi, M. Gyoda, T. Tabata, Y. Kuno, K. Yamazaki, M. Shibuya, Y. Seki and A. Yamazaki	2011	A considerate care robot able to serve in multi-party settings
284	Y. Kobayashi, Y. Kinpara, E. Takano, Y. Kuno, K. Yamazaki and A. Yamazaki	2011	Robotic Wheelchair Moving with Caregiver Collaboratively Depending on Circumstances
285	G. Koru, D. Alhuwail, M. Topaz, A. F. Norcio and M. E. Mills	2016	Investigating the Challenges and Opportunities in Home Care to Facilitate Effective Information Technology Adoption
286	N. M. Kosse, K. Brands, J. M. Bauer, T. Hortobagyi and C. J. C. Lamoth	2013	Sensor technologies aiming at fall prevention in institutionalized old adults: A synthesis of current knowledge
287	Y. Kowitlawakul	2011	The Technology Acceptance Model: Predicting Nurses' Intention to Use Telemedicine Technology (eICU)
288	Y. Kowitlawakul, S. W. C. Chan, J. Pulcini and W. Wang	2015	Factors influencing nursing students' acceptance of electronic health records for nursing education (EHRNE) software program
289	Y. Kowitlawakul, C. Moon Fai, S. Swee Lin Tan, A. Swee KitSoong and S. Wai Chi Chan	2017	Development of an e-Learning Research Module Using Multimedia Instruction Approach
290	A. J. Kozlowski, M. Fabian, D. Lad and A. D. Delgado	2017	Feasibility and Safety of a Powered Exoskeleton for Assisted Walking for Persons With Multiple Sclerosis: A Single-Group Preliminary Study
291	R. R. Kroll, J. G. Boyd and D. M. Maslove	2016	Accuracy of a wrist-Worn wearable device for monitoring heart rates in hospital inpatients:A prospective observational study

292	C. S. Kruse, M. Mileski, V. Alaytsev, E. Carol and A. Williams	2015	Adoption factors associated with electronic health record among long-term care facilities: a systematic review
293	G. Kumar, D. M. Falk, R. S. Bonello, J. M. Kahn, E. Perencevich and P. Cram	2013	The costs of critical care telemedicine programs: a systematic review and analysis
294	C. Kunkel, W. Kopp and M. Hanson	2016	A Matter of Life and Death: End-of-Life Simulation to Develop Confidence in Nursing Students
295	K.-M. Kuo, C.-F. Liu and C.-C. Ma	2013	An investigation of the effect of nurses? technology readiness on the acceptance of mobile electronic medical record systems
296	T. Kuroda, H. Noma, C. Naito, M. Tada, H. Yamanaka, T. Takemura, K. Nin and H. Yoshihara	2013	Prototyping sensor network system for automatic vital signs collection: Evaluation of a location based automated assignment of measured vital signs to patients
297	O. Kwon, J. M. Shim and G. Lim	2012	Single activity sensor-based ensemble analysis for health monitoring of solitary elderly people
298	T. Kymäläinen, J. Heinilä, T. Tuomisto, J. Plomp and T. Urhemaa	2012	Creating Scenes for an Intelligent Nursing Environment: Co-design and User Evaluations of a Home Control System
299	N. Laibhen-Parkes	2014	Web-Based evidence based practice educational intervention to improve EBP competence among BSN-prepared pediatric bedside nurses: A mixed methods pilot study
300	E. J. Lammers and C. G. McLaughlin	2017	Meaningful Use of Electronic Health Records and Medicare Expenditures: Evidence from a Panel Data Analysis of U.S. Health Care Markets, 2010-2013
301	R. L. N. Lang	2012	Evaluating the Effectiveness of Nurse-Focused Computerized Clinical Decision Support on Urinary Catheter Practice Guidelines
302	K. L. Lapane, C. M. Hughes, L. A. Daiello, K. A. Cameron and J. Feinberg	2011	Effect of a Pharmacist-Led Multicomponent Intervention Focusing on the Medication Monitoring Phase to Prevent Potential Adverse Drug Events in Nursing Homes
303	N. Lapierre, N. Neubauer, A. Miguel-Cruz, A. Rios Rincon, L. Liu and J. Rousseau	2018	The state of knowledge on technologies and their use for fall detection: A scoping review
304	S. Lapkin, T. Levett-Jones, L. Chenoweth and M. Johnson	2016	The effectiveness of interventions designed to reduce medication administration errors: a synthesis of findings from systematic reviews
305	A. S. M. Lau	2011	Hospital-based nurses' perceptions of the adoption of Web 2.0 tools for knowledge sharing, learning, social interaction and the production of collective intelligence

306	S. Lawn, X. Zhi and A. Morello	2017	An integrative review of e-learning in the delivery of self-management support training for health professionals
307	A. Lazar, G. Demiris and H. J. Thompson	2016	Evaluation of a multifunctional technology system in a memory care unit: Opportunities for innovation in dementia care
308	I. Lazarou, A. Karakostas, T. G. Stavropoulos, T. Tsompanidis, G. Meditskos, I. Kompatsiaris and M. Tsolaki	2016	A Novel and Intelligent Home Monitoring System for Care Support of Elders with Cognitive Impairment
309	C. L. Lear and C. Walters	2015	Use of Electronic Nurse Reminders to Improve Documentation
310	E. Lee	2015	Do Technology-Based Support Groups Reduce Care Burden Among Dementia Caregivers? A Review
311	S. J. Lee, S. S. Kim and Y. M. Park	2015	First experiences of high-fidelity simulation training in junior nursing students in Korea
312	S. Y. Lee and K. J. Cho	2017	A study on the cough augmentation using a belt-driven assistive
313	T. T. Lee and C. P. Chang	2016	Nurses' experiences of an initial and re-implemented electronic health record use
314	M. Leslie, E. Paradis, M. A. Gropper, S. Kitto, S. Reeves and P. Pronovost	2017	An Ethnographic Study of Health Information Technology Use in Three Intensive Care Units
315	M. Lexis	2013	Activity monitoring technology to support homecare delivery to frail and psychogeriatric elderly persons living at home alone
316	K. Li, S. Naganawa, K. Wang, P. Li, K. Kato, X. Li, J. Zhang and K. Yamauchi	2012	Study of the cost-benefit analysis of electronic medical record systems in general hospital in China
317	Y. Li, G. Chen, Y. Shen, Y. Zhu and Z. Cheng	2012	Accelerometer-based fall detection sensor system for the elderly
318	A. Liang, I. Piroth, H. Robinson, B. MacDonald, M. Fisher, U. M. Nater, N. Skoluda and E. Broadbent	2017	A Pilot Randomized Trial of a Companion Robot for People With Dementia Living in the Community
319	C. H. Liang, S. C. Chen, W. K. Lok, C. H. Luo and S. W. Lin	2011	A ZigBee-based electronic aid for daily living for quadriplegics
320	M. L. S. Lie, S. Lindsay and K. Brittain	2016	Technology and trust: Older people's perspectives of a home monitoring system
321	F. S. Lim, T. Wallace, M. A. Luszcz and K. J. Reynolds	2013	Usability of tablet computers by people with early-stage dementia
322	F. Lin, A. Wang, L. Cavuoto and W. Xu	2017	Toward Unobtrusive Patient Handling Activity Recognition for Injury Reduction among At-Risk Caregivers
323	H.-C. Lin	2017	Nurses' Satisfaction With Using Nursing Information Systems From Technology Acceptance Model and Information Systems Success Model Perspectives

324	H. H. Lin, Y. F. Chen, K. C. Lin and C. C. Yang	2014	Survey of satisfaction and usefulness for RFID-based clinical information system after system introduction
325	J. J. Lin, C. C. Lin and J. Wang	2011	Application of the electronic situated learning system for recognition of delirium in the elderly
326	Z.-C. Lin	2013	Comparison of technology-based cooperative learning with technology-based individual learning in enhancing fundamental nursing proficiency
327	L. Lind and D. Karlsson	2014	Telehealth for "the Digital Illiterate"-Elderly Heart Failure Patients Experiences
328	C. Lippincott	2014	The relationship between nursing excellence and electronic health record adoption
329	H. C. Liu, Y. J. Chen, Y. C. Lu, C. L. Wu, W. C. Huang and J. T. Huang	2013	Monitoring apnea in the elderly by an electromechanical system with a carbon Nanotube-based sensor
330	L. Liu, E. Stroulia, I. Nikolaidis, A. Miguel-Cruz and A. Rios Rincon	2016	Smart homes and home health monitoring technologies for older adults: A systematic review
331	C. C. Lo, T. Y. Chien, J. S. Pan and B. S. Lin	2016	Novel Non-Contact Control System for Medical Healthcare of Disabled Patients
332	Y. S. Lo, W. S. Lee, G. B. Chen and C. T. Liu	2014	Improving the work efficiency of healthcare-associated infection surveillance using electronic medical records
333	D. Lobach, G. D. Sanders, T. J. Bright, A. Wong, R. Dhurjati, E. Bristow, L. Bastian, R. Coeytaux, G. Samsa, V. Hasselblad, J. W. Williams, L. Wing, M. Musty and A. S. Kendrick	2012	Enabling health care decisionmaking through clinical decision support and knowledge management
334	M. Lobchuk, G. Halas, C. West, N. Harder, Z. Tursunova and C. Ramraj	2016	Development of a novel empathy-related video-feedback intervention to improve empathic accuracy of nursing students: A pilot study
335	A. Long, J. Edwards, R. Thompson, D. Lewis and A. Timoney	2014	A clinical evaluation of a sensor to detect blockage due to crystalline biofilm formation on indwelling urinary catheters
336	W. Y. G. Louie, D. McColl and G. Nejat	2012	Playing a memory game with a socially assistive robot: A case study at a long-term care facility
337	I. Lozano-Montoya, M. Vélez-Díaz-Pallarés, I. Abraha, A. Cherubini, R. L. Soiza, D. O'Mahony, B. Montero-Errasquín, A. Correa-Pérez and A. J. Cruz-Jentoft	2016	Nonpharmacologic Interventions to Prevent Pressure Ulcers in Older Patients: An Overview of Systematic Reviews (The Software ENgine for the Assessment and optimization of drug and non-drug Therapy in Older peRsons [SENATOR] Definition of Optimal Evidence-Based Non-drug Therapies in Older People [ONTOP] Series)

338	C.-H. Lu, J.-L. Hsiao and R.-F. Chen	2012	Factors determining nurse acceptance of hospital information systems
339	J. M. Luna, N. Yip, R. Pivovarov and D. K. Vawdrey	2016	Representativeness comparisons of nurse and computer charting of heart rate across nursing-intensity protocols
340	M. Lyngstad, D. Hofoss, A. Grimsmo and R. Hellesø	2015	Predictors for assessing electronic messaging between nurses and general practitioners as a useful tool for communication in home health care services: A cross-sectional study
341	B. Lyons, A. Lindauer, A. Seelye, K. Mincks, J. Kaye and D. Erten-Lyons	2016	Distance and intimacy: an ethnographic analysis of the strengths and limitations of video telemedicine care for dementia
342	S. Macis, D. Loi and L. Raffo	2016	The HEREiAM Tele-social-care Platform for Collaborative Management of Independent Living
343	K. Maclure, D. Stewart and A. Strath	2014	A systematic review of medical and non-medical practitioners' views of the impact of ehealth on shared care
344	K. Madara Marasinghe	2016	Assistive technologies in reducing caregiver burden among informal caregivers of older adults: a systematic review
345	Y. Maekawa, Y. Akiyama and S. Nishijima	2011	Development of an accident detection system for care service users by image and motion analysis
346	D. L. Magtibay, S. S. Chesak, K. Coughlin and A. Sood	2017	Decreasing Stress and Burnout in Nurses: Efficacy of Blended Learning With Stress Management and Resilience Training Program
347	M. Mahdavian, H. Nazarian, M. Mahdavian and N. Wattanapongsakorn	2014	An investigation of the success of hospital information systems implementation: A case study
348	É. Maillet, L. Mathieu and C. Sicotte	2015	Modeling factors explaining the acceptance, actual use and satisfaction of nurses using an Electronic Patient Record in acute care settings: An extension of the UTAUT
349	V. Makkapati, P. Raman and G. Pai	2016	Camera based respiration rate of neonates by modeling movement of chest and abdomen region
350	A. Mammen and R. Weeks	2014	Electronic Medical Record (EMR) technology acceptance by healthcare professionals in South Africa
351	E. Manias, A. Williams and D. Liew	2012	Interventions to reduce medication errors in adult intensive care: A systematic review
352	S. Manimaran and K. B. Lakshmi	2013	Development of model for assessing the acceptance level of users in rural healthcare system of Tamilnadu, India

353	S. Manrique-Rodríguez, A. Sánchez-Galindo, C. M. Fernández-Llamazares, J. López-Herce, L. Echarri-Martínez, V. Escudero-Vilaplana, M. Sanjuro-Sáez and Á. Carrillo-álvarez	2012	Smart pump alerts: All that glitters is not gold
354	S. Manrique-Rodríguez, A. C. Sánchez-Galindo, A. de Lorenzo-Pinto, L. González-Vives, J. López-Herce, Á. Carrillo-Álvarez, M. Sanjurjo-Sáez and C. M. Fernández-Llamazares	2015	Implementation of smart pump technology in a paediatric intensive care unit
355	S. Manrique-Rodríguez, A. C. Sánchez-Galindo, J. López-Herce, M. Á. Calleja-Hernández, F. Martínez-Martínez, I. Iglesias-Peinado, Á. Carrillo-Álvarez, M. Sanjurjo-Sáez and C. M. Fernández-Llamazares	2014	Implementing smart pump technology in a pediatric intensive care unit: A cost-effective approach
356	J. Mansfield and S. Jarrett	2013	Original article: Using smart pumps to understand and evaluate clinician practice patterns to ensure patient safety
357	K. M. Marasinghe	2015	Computerised clinical decision support systems to improve medication safety in long-term care homes: A systematic review
358	K. Marek, F. Stetzer, P. Ryan, L. Bub, S. Adams, A. Schlidt, R. Lancaster and A. O'Brien	2013	Nurse care coordination and technology effects on health status of frail older adults via enhanced self-management of medication: randomized clinical trial to test efficacy
359	K. D. Marek, F. Stetzer, S. J. Adams, L. D. Bub, A. Schlidt and K. J. Colorafi	2014	Cost Analysis of a Home-Based Nurse Care Coordination Program
360	E. Markowitz	2011	Evaluating the usability of the OpenVista Electronic Health Record EHR
361	A. R. Marra, T. Z. Sampaio Camargo, T. P. Magnus, R. P. Blaya, G. B. dos Santos, L. R. Guastelli, R. D. Rodrigues, M. Prado, E. d. S. Victor, H. Bogossian, J. C. M. Monte, O. F. P. o. dos Santos, C. K. Oyama and M. B. Edmond	2014	The use of real-time feedback via wireless technology to improve hand hygiene compliance

362	M. Marschollek, M. Becker, J. M. Bauer, P. Bente, L. Dasenbrock, K. Elbers, A. Hein, G. Kolb, H. Künemund, C. Lammel-Polchau, M. Meis, H. Meyer Zu Schwabedissen, H. Remmers, M. Schulze, E.-E. Steen, W. Thoben, J. Wang, K.-H. Wolf and R. Haux	2014	Multimodal activity monitoring for home rehabilitation of geriatric fracture patients - feasibility and acceptance of sensor systems in the GAL-NATARS study
363	M. Marschollek, A. Rehwald, K. H. Wolf, M. Gietzelt, G. Nemitz, H. M. Zu Schwabedissen and M. Schulze	2011	Sensors vs. experts - A performance comparison of sensor-based fall risk assessment vs. conventional assessment in a sample of geriatric patients
364	S. Martin, E. Armstrong and J. Daly	2015	A Brain Computer Interface to support independence and function of people with acquired brain injury living at home...39th annual conference and exhibition of the College of Occupational Therapists, Brighton and Sussex, England. June 30-July 2, 2015
365	A. Martínez, C. I. Ramirez-Salvador, A. José, R. L. Alejandra and B. Jiménez-Rodríguez	2017	An eService platform for the assistance and support of primary caregivers
366	R. Martinez-Maldonado, M. Pechenizkiy, S. Buckingham Shum, T. Power, C. Hayes and C. Axisa	2017	Modelling Embodied Mobility Teamwork Strategies in a Simulation-Based Healthcare Classroom
367	J. J. Mason, R. e. Roberts-Turner, V. Amendola, A. M. Sill and P. S. Hinds	2014	Patient Safety, Error Reduction, and Pediatric Nurses' Perceptions of Smart Pump Technology
368	S. D. Mazzacano, T. McSherry, M. Atterbury, E. Helmold, S. Gartner and C. Schulman	2016	Effect of virtual reality distraction therapy on pain and anxiety in adult patients undergoing complex dressing changes: a randomized controlled trial
369	S. McBride, M. Tietze, M. A. Hanley and L. Thomas	2017	Statewide Study to Assess Nurses' Experiences With Meaningful Use-Based Electronic Health Records
370	T. McDonald and F. Russell	2012	Impact of technology-based care and management systems on aged care outcomes in Australia
371	M. McGuckin and J. Govednik	2015	A Review of Electronic Hand Hygiene Monitoring: Considerations for Hospital Management in Data Collection, Healthcare Worker Supervision, and Patient Perception
372	M. S. McHenry, L. J. Fischer, Y. Chun and R. C. Vreeman	2017	A systematic review of portable electronic technology for health education in resource-limited settings
373	R. M. McKenna, D. Dwyer and J. A. Rizzo	2017	Is HIT a hit? The impact of health information technology on inpatient hospital outcomes

374	K. A. McKibbon, C. Lokker, S. M. Handler, L. R. Dolovich, A. M. Holbrook, D. O'Reilly, R. Tamblyn, J. H. B, R. Basu, S. Troyan, P. S. Roshanov, N. P. Archer and P. Raina	2011	Enabling medication management through health information technology (Health IT)
375	K. A. McKibbon, C. Lokker, S. M. Handler, L. R. Dolovich, A. M. Holbrook, D. O'Reilly, R. Tamblyn, B. J. Hemens, R. Basu, S. Troyan, P. S. Roshanov, K. A. McKibbon, C. Lokker, S. M. Handler, L. R. Dolovich, A. M. Holbrook, D. O'Reilly, R. Tamblyn, B. J. Hemens and R. Basu	2012	The effectiveness of integrated health information technologies across the phases of medication management: a systematic review of randomized controlled trials
376	R. Meehan	2017	Electronic Health Records in Long-Term Care: Staff Perspectives
377	S. Mehner, R. Klauck and H. Koenig	2013	Location-independent Fall Detection with Smartphone
378	Y. Y. Mei, J. Marquard, C. Jacelon and A. L. DeFeo	2013	Designing and evaluating an electronic patient falls reporting system: Perspectives for the implementation of health information technology in long-term residential care facilities
379	A. Meißner and W. Schnepp	2014	Staff experiences within the implementation of computer-based nursing records in residential aged care facilities: a systematic review and synthesis of qualitative research
380	L. Melby, B. J. Brattheim and R. Hellesø	2015	Patients in transition - improving hospital-home care collaboration through electronic messaging: providers' perspectives
381	S. Merilampi, A. Koivisto, A. Sirkka, P. Raumonen, J. Virkki, X. Xiao, Y. Min, L. Ye, X. Chujun and J. Chen	2017	The cognitive mobile games for older adults-A Chinese user experience study
382	S. Meyer, R. G. Heinze, M. Neitzel, M. Sudau and C. Wedemeier	2015	Technische Assistenzsysteme für ältere Menschen - eine Zukunftsstrategie für die Bau- und Wohnungswirtschaft Wohnen für ein langes Leben/AAL
383	C. Meyer-Delpho and H. J. Schubert	2014	Potential of Information and Communications Technology to Improve Intersectoral Processes of Care: A Case Study of the Specialised Outpatient Palliative Care
384	K. Mi Ok, E. Coiera, F. Magrabi and M. O. Kim	2017	Problems with health information technology and their effects on care delivery and patient outcomes: a systematic review
385	M. B. Michel-Verkerke and A. M. G. M. Hoogeboom	2012	Evaluation of an Electronic Patient Record in a Nursing Home: One Size Fits All?

386	S. Mickan, H. Atherton, N. W. Roberts, C. Heneghan and J. K. Tilson	2014	Use of handheld computers in clinical practice: A systematic review
387	L. Mierlo, F. Meiland, P. Ven, H. Hout and R. Dröes	2015	Evaluation of DEM-DISC, customized e-advice on health and social support services for informal carers and case managers of people with dementia; a cluster randomized trial
388	K. Miller, S. Rodger, B. Kipping and R. M. Kimble	2011	A novel technology approach to pain management in children with burns: A prospective randomized controlled trial
389	A. Mishra, S. Rani and U. D. Bhardwaj	2017	Effectiveness of E-learning Module on First Aid: A Study on Student Nurses
390	S. Mitchell and U. Yaylacicegi	2012	EHR prescription for small, medium, and large hospitals: an exploratory study of Texas acute care hospitals
391	M. Mittelman, C. Epstein and J. Hobday	2015	Efficacy of internet-based training of clinicians to implement an evidence-based intervention for dementia caregivers
392	T. L. Mitzner, C. C. Kemp, W. Rogers and L. Tiberio	2013	Investigating Healthcare Providers? Acceptance of Personal Robots for Assisting with Daily Caregiving Tasks
393	T. Miyoshi, H. Yamazoe and J. H. Lee	2015	Natural behavior based teleoperation for dual robot manipulators mounted on a wheelchair
394	E. Mlaver, J. L. Schnipper, R. B. Boxer, D. J. Breuer, E. F. Gershanik, P. C. Dykes, A. F. Massaro, J. Benneyan, D. W. Bates and L. S. Lehmann	2017	User-Centered Collaborative Design and Development of an Inpatient Safety Dashboard
395	J. Moeckli, P. Cram, C. Cunningham and H. S. Reisinger	2013	Staff acceptance of a telemedicine intensive care unit program: A qualitative study
396	S. Mohapatra and S. Murarka	2016	Improving patient care in hospital in India by monitoring influential parameters
397	C. Mollaret, A. A. Mekonnen, F. Lerasle, I. Ferrané, J. Pinquier, B. Boudet and P. Rumeau	2016	A multi-modal perception based assistive robotic system for the elderly
398	L. Montanini, L. Raffaeli, A. De Santis, A. Del Campo, C. Chiatti, G. Rascioni, E. Gambi and S. Spinsante	2016	Overnight supervision of Alzheimer's disease patients in nursing homes: System development and field trial
399	S. M. Montenery, M. Walker, E. Sorensen, R. Thompson, D. Kirklin, R. White and C. Ross	2013	Millennial Generation Student Nurses' Perceptions of the Impact of Multiple Technologies on Learning
400	L. M. Monti Fonseca, N. Del' Angelo Aredes, A. M. Fernandes, L. M. da Cunha Batalha, J. M. Amado Apóstolo, J. C. Amado Martins and M. Alves Rodrigues	2016	Computer and laboratory simulation in the teaching of neonatal nursing: innovation and impact on learning

401	S. S. Moreland, M. L. Lemieux and A. Myers	2012	End-of-life Care and the Use of Simulation in a Baccalaureate Nursing Program
402	L. Morente, J. M. Morales-Asencio and F. J. Veredas	2014	Effectiveness of an e-learning tool for education on pressure ulcer evaluation
403	M. J. Morón, R. Yáñez, D. Cascado, C. Suárez-Mejías and J. L. Sevillano	2014	A mobile memory game for patients with Acquired Brain Damage: A preliminary usability study
404	L. Mosalanejad, S. Shahsavari, S. Sobhanian and M. Dastpak	2012	The effect of virtual versus traditional learning in achieving competency-based skills
405	P. Moule, R. Ward and L. Lockyer	2011	Issues with e-learning in nursing and health education in the uk: Are new technologies being embraced in the teaching and learning environments?
406	W. Moyle, U. Arnautovska, T. Ownsworth and C. Jones	2017	Potential of telepresence robots to enhance social connectedness in older adults with dementia: an integrative review of feasibility
407	W. Moyle, M. Bramble, C. Jones and J. Murfield	2016	Care staff perceptions of a social robot called Paro and a look-alike Plush Toy: a descriptive qualitative approach
408	W. Moyle, M. Cooke, E. Beattie, C. Jones, B. Klein, G. Cook and C. Gray	2013	Exploring the effect of companion robots on emotional expression in older adults with dementia: a pilot randomized controlled trial
409	W. Moyle, C. Jones, M. Cooke, S. O. Dwyer, B. Sung and S. Drummond	2013	Social robots helping people with dementia: Assessing efficacy of social robots in the nursing home environment
410	W. Moyle, C. Jones, M. Cooke, S. O'Dwyer, B. Sung and S. Drummond	2014	Connecting the person with dementia and family: A feasibility study of a telepresence robot
411	W. Moyle, C. J. Jones, J. E. Murfield, L. Thalib, E. R. A. Beattie, D. K. H. Shum, S. T. O'Dwyer, M. C. Mervin and B. M. Draper	2017	Use of a Robotic Seal as a Therapeutic Tool to Improve Dementia Symptoms: A Cluster-Randomized Controlled Trial
412	S. Mu, S. Nakashima and K. Tanaka	2014	Applications of ultrasonic sensors and obrid-sensor in safety confirmation system for elders
413	F. Muheidat and H. W. Tyrer	2017	Deriving Information from Low Spatial Resolution Floor-Based Personnel Detection System
414	M. Mullen-Fortino, J. DiMartino, L. Entrikin, S. Mulliner, C. W. Hanson and J. M. Kahn	2012	BEDSIDE NURSES' PERCEPTIONS OF INTENSIVE CARE UNIT TELEMEDICINE
415	L. Müller, M. Sonnentag and S. Heuer	2013	Supporting reflection on dementia care using proximity sensors
416	W. C. Mundell, C. C. Kennedy, J. H. Szostek and D. A. Cook	2013	Simulation technology for resuscitation training: A systematic review and meta-analysis

417	M. Munstermann	2015	Technisch unterstützte Pflege von morgen - innovative Aktivitätserkennung und Verhaltensermittlung durch ambiente Sensorik
418	E. N. Munyisia, P. Yu and D. Hailey	2011	The changes in caregivers' perceptions about the quality of information and benefits of nursing documentation associated with the introduction of an electronic documentation system in a nursing home
419	E. N. Munyisia, P. Yu and D. Hailey	2012	The impact of an electronic nursing documentation system on efficiency of documentation by caregivers in a residential aged care facility
420	E. Murray, J. Burns, C. May, T. Finch, C. O'Donnell, P. Wallace and F. Mair	2011	Why is it difficult to implement e-health initiatives? A qualitative study
421	R. Musanti, M. Downing, D. A. Forrester, D. Fochesto and P. O'Keefe	2015	Staff perceptions of patient visibility systems in acute care settings
422	M. B. Muthuswamy, B. N. Thomas, D. Williams and J. Dingley	2014	Utility of optical facial feature and arm movement tracking systems to enable text communication in critically ill patients who cannot otherwise communicate
423	A. Nagata, Z. Huang, M. Kanai-Pak, J. Maeda, Y. Kitajima, M. Nakamura, K. Aida, N. Kuwahara, T. Ogata and J. Ota	2012	Supporting system for self training of bed-making using image processing with color and distance information
424	A. Nagler, J. Schlueter, C. Johnson, B. Griffith, J. Prewitt, R. Sloane and M. Adams	2014	Calling for collaboration: piloting smartphones to discover differences between users and devices
425	S. Nakagawa, D. P, J. Huang, K. Sekiyama and T. Fukuda	2013	Control of intelligent cane robot considering usage of ordinary cane
426	S. Nakrem, M. Solbjør, I. N. Pettersen and H. H. Kleiven	2018	Care relationships at stake? Home healthcare professionals' experiences with digital medicine dispensers - A qualitative study
427	M. Namnabati, F. Taleghani, M. Varzeshnejad, A. Yousefi, Z. Karjoo and S. Safiri	2017	Nursing care and documentation assistant with an electronic nursing management system in neonatal intensive care unit
428	R. F. Navarro and J. Favela	2011	Usability Assessment of a Pervasive System to Assist Caregivers in Dealing with Repetitive Behaviors of Patients with Dementia
429	T. Nef, P. Urwyler, M. Büchler, I. Tarnanas, R. Stucki, D. Cazzoli, R. Müri and U. Mosimann	2012	Evaluation of Three State-of-the-Art Classifiers for Recognition of Activities of Daily Living from Smart Home Ambient Data
430	M. Neggazi, L. Hamami and A. Amira	2014	Efficient compressive sensing on the shimmer platform for fall detection

431	C. P. Nemeth, J. Brown, B. Crandall and C. Fallon	2014	The mixed blessings of smart infusion devices and health care IT
432	J. Neuhaeuser, J. Diehl-Schmid and T. C. Lueth	2011	Evaluation of a radio based ADL interaction recognition system in a day hospital for old age psychiatry with healthy probands
433	B. B. Neves, R. L. Franz, C. Munteanu, R. Baecker and M. Ngo	2015	My Hand Doesn?T Listen to Me!: Adoption and Evaluation of a Communication Technology for the ?Oldest Old?
434	L. Nguyen, E. Bellucci and L. T. Nguyen	2014	Electronic health records implementation: An evaluation of information system impact and contingency factors
435	L. Nguyen, N. Wickramasinghe, B. Redley, P. Haddad, I. Muhammad and M. Botti	2017	Exploring nurses' reactions to electronic nursing documentation at the point of care
436	B. Ni, C. D. Nguyen and P. Moulin	2012	RGBD-camera based get-up event detection for hospital fall prevention
437	A. Nicolson, L. Moir and J. Millsteed	2012	Impact of assistive technology on family caregivers of children with physical disabilities: a systematic review
438	N. Nijhof, J. E. W. C. van Gemert-Pijnen, C. M. Burns and E. R. Seydel	2013	A personal assistant for dementia to stay at home safe at reduced cost
439	N. Nijhof, L. J. van Gemert-Pijnen, R. Woolrych and A. Sixsmith	2013	An evaluation of preventive sensor technology for dementia care
440	T. Nilpanapan and T. Kerdcharoen	2017	Social data shoes for gait monitoring of elderly people in smart home
441	H. E. Nilsson, J. Sidén and M. Gulliksson	2011	An incontinence alarm solution utilizing RFID based sensor technology
442	J. Nordheim, S. Hamm, A. Kuhlmey and R. Suhr	2015	Tablet computers and their benefits for nursing home residents with dementia: Results of a qualitative pilot study
443	L. L. Novak, S. Anders, C. S. Gadd, N. M. Lorenzi, L. L. Novak, S. Anders, C. S. Gadd and N. M. Lorenzi	2012	Mediation of adoption and use: a key strategy for mitigating unintended consequences of health IT implementation
444	B. T. Nukala, N. Shibuya, A. I. Rodriguez, J. Tsay, T. Q. Nguyen, S. Zupancic and D. Y. C. Lie	2014	A real-time robust fall detection system using a wireless gait analysis sensor and an Artificial Neural Network
445	B. Oakley and J. B. Hunter	2017	Implementing an electronic patient handover system
446	A. Odunmbaku, A. M. Rahmani, P. Liljeberg and H. Tenhunen	2016	Elderly monitoring system with sleep and fall detector
447	N. Ofek Shlomai, S. Rao and S. Patole	2015	Efficacy of interventions to improve hand hygiene compliance in neonatal units: a systematic review and meta-analysis
448	T. Ogura, T. Itami, K. Yano, I. Mori and K. Kameda	2017	An assistance device to help people with trunk impairment maintain posture

449	T. Ohiwa, H. Yamamoto and K. Yamazaki	2016	Sensor system to advice health-aware information for elderly people on daily living activities
450	T. Okano, D. Kitakoshi and M. Suzuki	2013	A Preliminary Study on Preventive Care System Based on Game Playing with Communication Robots
451	B. Okoniewska, A. Graham, M. Gavrilova, D. Wah, J. Gilgen, J. Coke, J. Burden, S. Nayyar, J. Kaunda, D. Yergens, B. Baylis and W. A. Ghali	2012	Multidimensional evaluation of a radio frequency identification wi-fi location tracking system in an acute-care hospital setting
452	N. Olchanski, M. A. Dziadzko, I. C. Tiong, C. E. Daniels, S. G. Peters, J. C. O'Horo and M. N. Gong	2017	Can a Novel ICU Data Display Positively Affect Patient Outcomes and Save Lives?
453	D. P. Oliver, G. Demiris, E. Wittenberg-Lyles, K. Washington, T. Day and H. Novak	2012	A systematic review of the evidence base for telehospice
454	C. K. Or, B. T. Karsh, D. J. Severtson, L. J. Burke, R. L. Brown, P. F. Brennan, C. K. L. Or, B.-T. Karsh, D. J. Severtson, L. J. Burke, R. L. Brown and P. F. Brennan	2011	Factors affecting home care patients' acceptance of a web-based interactive self-management technology
455	P. Ordonez, T. Oates, M. E. Lombardi, G. Hernandez, K. W. Holmes, J. Fackler and C. U. Lehmann	2012	Visualization of multivariate time-series data in a neonatal ICU
456	E. M. Orellano-Colón, W. C. Mann, M. Rivero, M. Torres, J. Jutai, A. Santiago and N. Varas	2016	Hispanic older adult's perceptions of personal, contextual and technology-related barriers for using assistive technology devices
457	V. Orto, C. C. Hendrix, B. Griffith and S. T. Shaikewitz	2015	Implementation of a Smart Pump Champions Program to Decrease Potential Patient Harm
458	A. A. A. Osaimi, K. A. Kadi and B. Saddik	2017	Role of radio frequency identification in improving infant safety and the extent of nursing staff acceptance of RFID at King Abdulaziz medical city in Riyadh
459	Y. Ouyang, K. Shan and F. M. Bui	2016	An RF-based wearable sensor system for indoor tracking to facilitate efficient healthcare management
460	J. Y. Pai, D. Liu, I. H. Lin and H. C. Lai	2017	Apply Information and Communications Technology to Improve the Quality of Day Care Center
461	E. Palm	2013	An interactive ethical assessment of surveillance-capable software within the home-help service sector

462	C. Panagopoulos, E. Kalatha, P. Tsanakas and I. Maglogiannis	2015	Evaluation of a mobile home care platform lessons learned and practical guidelines
463	D. Panzoli, C. Pons-Lelardeux and P. Lagarrigue	2015	Communication and Knowledge Sharing in an Immersive Learning Game
464	P. A. Pappas, L. Tirelli, J. Shaffer and S. Gettings	2016	Projecting Critical Care Beyond the ICU: An Analysis of Tele-ICU Support for Rapid Response Teams
465	S. O. Paranilam	2013	Effectiveness of an Electronic Pain Notification System on Postoperative Pain
466	G. Paré, P. Poba-Nzaou and C. Sicotte	2013	Home telemonitoring for chronic disease management: An economic assessment
467	G. Pare, C. Sicotte, M. P. Moreault, P. Poba-Nzaou, M. Templier and G. Nahas	2011	Effects of Mobile Computing on the Quality of Homecare Nursing Practice
468	C. Park, S. Kang, J. Kim and J. Oh	2012	A study on service robot system for elder care
469	J. L. Partin, T. A. Payne and M. F. Slemmons	2011	Students' perceptions of their learning experiences using high-fidelity simulation to teach concepts relative to obstetrics
470	F. L. Patmon, P. M. Gee, T. L. Rylee and N. L. Readdy	2016	Using Interactive Patient Engagement Technology in Clinical Practice: A Qualitative Assessment of Nurses' Perceptions
471	D. Patterson, M. Soltani, A. Teeley, D. Morse, S. Wiechman and N. Gibran	2012	Hypnosis delivered through immersive virtual reality for wound care: a randomized, controlled study
472	A. J. Pearce, B. Adair, K. Miller, E. Ozanne, C. Said, N. Santamaria and M. E. Morris	2012	Robotics to enable older adults to remain living at home
473	S. T. M. Peek, E. J. M. Wouters, J. van Hoof, K. G. Luijkx, H. R. Boeije and H. J. M. Vrijhoef	2014	Factors influencing acceptance of technology for aging in place: A systematic review
474	J. M. Peeters, A. J. E. de Veer, L. van der Hoek and A. L. Francke	2012	Factors influencing the adoption of home telecare by elderly or chronically ill people: a national survey
475	J. M. Peeters, T. A. Wiegers and R. D. Friele	2013	How technology in care at home affects patient self-care and self-management: A scoping review
476	V. Pemmassani, T. Paget, H. C. van Woerden and S. Pemmasani	2014	Hands-free communication to free up nursing time
477	N. Peng, R. Zhang, H. Zeng, F. Wang, K. Li, Y. Li and X. Zhuang	2016	Control of a nursing bed based on a hybrid brain-computer interface
478	P. Perego, G. Andreoni, R. Zanini and R. Bellù	2014	Wearable biosignal monitoring system for newborns
479	H. H. C. Peres, A. F. C. Lima, D. d. A. L. M. d. Cruz, R. R. Gaidzinski, N. B. Oliveira, D. C. F. Ortiz, M. M. e. Trindade and R. Tsukamoto	2012	Assessment of an electronic system for clinical nursing documentation

480	D. Peretz, A. Arnaert and N. N. Ponzoni	2018	Determining the cost of implementing and operating a remote patient monitoring programme for the elderly with chronic conditions: A systematic review of economic evaluations
481	M. B. Perrigino and B. B. Dunford	2016	The unique effects of general and specific support in health care technology: An empirical examination of the principle of compatibility
482	S. Perri-Moore, S. Kapsandoy, K. Doyon, B. Hill, M. Archer, L. Shane-McWhorter, B. E. Bray and Q. Zeng-Treitler	2016	Automated alerts and reminders targeting patients: A review of the literature
483	C. Peters, T. Hermann, S. Wachsmuth and J. Hoey	2014	Automatic Task Assistance for People with Cognitive Disabilities in Brushing Teeth - A User Study with the TEBRA System
484	S. Petersen, S. Houston, H. Qin, C. Tague and J. Studley	2017	The Utilization of Robotic Pets in Dementia Care
485	J. Petty	2013	Interactive, technology-enhanced self-regulated learning tools in healthcare education: A literature review
486	N. Philipsen, W. Carruthers, G. Chi, D. Ensey, A. Shmorhun and R. Valdez	2014	A mixed-methods assessment of time spent documenting by nurses using an electronic medical records system
487	D. Pickham, N. Berte, M. Pihulic, A. Valdez, B. Mayer and M. Desai	2018	Effect of a wearable patient sensor on care delivery for preventing pressure injuries in acutely ill adults: A pragmatic randomized clinical trial (LS-HAPI study)
488	P. Pierleoni, A. Belli, L. Palma, L. Pernini and S. Valenti	2014	A versatile ankle-mounted fall detection device based on attitude heading systems
489	L. Pigini, G. Bovi, C. Panzarino, V. Gower, M. Ferratini, G. Andreoni, R. Sassi, M. W. Rivolta and M. Ferrarin	2017	Pilot Test of a New Personal Health System Integrating Environmental and Wearable Sensors for Telemonitoring and Care of Elderly People at Home (SMARTA Project)
490	H. Pigot, P. Y. Nivollet, T. Zayani, Y. Adelise and L. Domus	2016	Ubiquitous reminders to manage timetable in a smart home
491	L. L. Pineles, D. J. Morgan, H. M. Limper, S. G. Weber, K. A. Thom, E. N. Perencevich, A. D. Harris and E. Landon	2014	Accuracy of a radiofrequency identification (RFID) badge system to monitor hand hygiene behavior during routine clinical activities
492	M. Pino, M. Boulay, F. Jouen and A. S. Rigaud	2015	Are we ready for robots that care for us? Attitudes and opinions of older adults toward socially assistive robots
493	K. Pitts, K. Pudney, K. Zachos, N. Maiden, B. Krogstie, S. Jones, M. Rose, J. MacManus and I. Turner	2015	Using mobile devices and apps to support reflective learning about older people with dementia
494	A. C. Polycarpou, A. Dimitriou, A. Bletsas, P. C. Polycarpou, L. Papaloizou, G. Gregoriou and J. N. Sahalos	2012	On the Design, Installation, and Evaluation of a Radio-Frequency Identification System for Healthcare Applications [Wireless Corner]

495	F. Portela, M. F. Santos, J. Machado, A. Abelha, J. Neves, A. Silva and F. Rua	2012	Intelligent decision support in intensive care - Towards technology acceptance
496	F. R. Portela, R. J. C. Correia, J. A. Fonseca and J. M. Andrade	2011	Wiitherapy on seniors — Effects on physical and metal domains
497	A. M. Pot, B. M. Willemse and S. Horjus	2012	A pilot study on the use of tracking technology: Feasibility, acceptability, and benefits for people in early stages of dementia and their informal caregivers
498	J. Powers	2016	Two Methods for Turning and Positioning and the Effect on Pressure Ulcer Development: a Comparison Cohort Study
499	J. Pripfl, T. Kortner, D. Batko-Klein, D. Hebesberger, M. Weninger and C. Gisinger	2016	Social service robots to support independent living : Experiences from a field trial
500	G. W. Pritchard and K. Brittain	2015	Alarm pendants and the technological shaping of older people's care. Between (intentional) help and (irrational) nuisance
501	S. Qian and P. Yu	2014	Fitting clinical workflow: The case for wound care in a residential aged care home
502	W. Quan, H. Niwa, N. Ishikawa, Y. Kobayashi and Y. Kuno	2011	Assisted-care robot based on sociological interaction analysis
503	A. Queirós, A. Silva, J. Alvarelhão, N. P. Rocha and A. Teixeira	2015	Usability, accessibility and ambient-assisted living: a systematic literature review
504	K. Radhakrishnan, B. Xie, A. Berkley and M. Kim	2016	Barriers and Facilitators for Sustainability of Tele-Homecare Programs: A Systematic Review
505	J. Raman	2015	Mobile technology in nursing education: where do we go from here? A review of the literature
506	V. R. Ramnath and N. Khazeni	2014	Centralized monitoring and virtual consultant models of tele-ICU care: a side-by-side review
507	R. Ranasinghe, L. Dantanarayana, A. Tran, S. Lie, M. Behrens and L. Liu	2014	Smart hoist: An assistive robot to aid carers
508	P. Rantanen, T. Parkkari, S. Leikola, M. Airaksinen and A. Lyles	2017	An In-Home Advanced Robotic System to Manage Elderly Home-Care Patients' Medications: a Pilot Safety and Usability Study
509	M. Rantz, L. J. Phillips, C. Galambos, K. Lane, G. L. Alexander, L. Despins, R. J. Koopman, M. Skubic, L. Hicks, S. Miller, A. Craver, B. H. Harris and C. B. Deroche	2017	Randomized Trial of Intelligent Sensor System for Early Illness Alerts in Senior Housing

510	M. J. Rantz, G. Alexander, C. Galambos, M. K. Flesner, A. Vogelsmeier, L. Hicks, J. Scott-Cawiezell, M. Zwygart-Stauffacher and L. Greenwald	2011	The use of bedside electronic medical record to improve quality of care in nursing facilities: a qualitative analysis
511	P. Ray, J. Li, A. Ariani and V. Kapadia	2017	Tablet-Based Well-Being Check for the Elderly: Development and Evaluation of Usability and Acceptability
512	S. J. Redmond, Z. Zhang, M. R. Narayanan and N. H. Lovell	2014	Pilot evaluation of an unobtrusive system to detect falls at nighttime
513	C. Reed, D. Fraley, J. Sharp, A. Hernandez, J. Proulx, A. Kattan, H. Williams and R. Wilson	2013	Video instruction increases accuracy in performance of nurse dysphagia screening
514	S. C. Reed, K. Kim, V. Ngo, W. Wait, E. Apesoa-Varano, J. Morgan, R. L. Whitney, E. Blackmon and J. Joseph	2016	Usability and acceptance of novel personal health technology to support early palliative care for patients with cancer and caregivers
515	B. Reeder, G. Demiris and K. Marek	2013	Older adults' satisfaction with a medication dispensing device in home care
516	I. T. Reierson, H. Solli and I. T. Bjørk	2015	Nursing students' perspectives on telenursing in patient care after simulation
517	Z. S. N. Reis, T. A. Maia, M. S. Marcolino, F. Becerra-Posada, D. Novillo-Ortiz and A. L. P. Ribeiro	2017	Is There Evidence of Cost Benefits of Electronic Medical Records, Standards, or Interoperability in Hospital Information Systems? Overview of Systematic Reviews
518	L. Ren, Q. Zhang and W. Shi	2012	Low-power Fall Detection in Home-based Environments
519	J. D. Restuccia, A. B. Cohen, J. N. Horwitt and M. Shwartz	2012	Hospital implementation of health information technology and quality of care: Are they related?
520	C. Ribeiro, M. Monteiro, J. B. Hauge, J. Pereira and T. Antunes	2016	Sepsis Fast Track: A simulation game for Clinical education based on the Sepsis Fast Track protocol
521	J. Richter, C. Wiede, E. Dayangac, A. Shahenshah and G. Hirtz	2017	Activity recognition for elderly care by evaluating proximity to objects and human skeleton data
522	J. River, J. Currie, T. Crawford, V. Betihavas and S. Randall	2016	A systematic review examining the effectiveness of blending technology with team-based learning
523	S. Robben, L. Bosch, P. Wiggers, J. Decancq and M. Kanis	2015	Managing Flexible Care with a Context Aware System for Ageing-in-place
524	P. Robert, E. Castelli, P. C. Chung, T. Chiroux, C. F. Crispim-Junior, P. Mallea and F. Bremond	2013	SWEET-HOME ICT technologies for the assessment of elderly subjects
525	S. Roberts, A. Marshall and W. Chaboyer	2017	Hospital staffs' perceptions of an electronic program to engage patients in nutrition care at the bedside: A qualitative study

526	S. Roberts, A. P. Marshall, R. Gonzalez and W. Chaboyer	2017	Technology to engage hospitalised patients in their nutrition care: a qualitative study of usability and patient perceptions of an electronic foodservice system
527	E. J. Robinson, M. R. Bergey, E. Brady, A. M. Mapp and J. C. Goldsack	2017	The Impact of an Electronic Medication Administration Record (eMAR) and Computerized Physician Order Entry (CPOE) on Nurse Extender and Unit Clerk Staffing
528	H. Robinson, B. MacDonald and E. Broadbent	2015	Physiological effects of a companion robot on blood pressure of older people in residential care facility: a pilot study
529	H. Robinson, B. MacDonald, N. Kerse and E. Broadbent	2013	The Psychosocial Effects of a Companion Robot: A Randomized Controlled Trial
530	H. Robinson, B. A. MacDonald, N. Kerse and E. Broadbent	2013	Suitability of Healthcare Robots for a Dementia Unit and Suggested Improvements
531	L. Robinson, G. Gibson, A. Kingston, L. Newton, G. Pritchard, T. Finch and K. Brittain	2013	Assistive technologies in caring for the oldest old: A review of current practice and future directions
532	C. S. Rodriguez	2016	ENHANCING THE COMMUNICATION OF SUDDENLY SPEECHLESS CRITICAL CARE PATIENTS
533	P. S. Roshanov, N. Fernandes, J. M. Wilczynski, B. J. Hemens, J. J. You, S. M. Handler, R. Nieuwlaat, N. M. Souza, J. Beyene, H. G. C. Van Spall, A. X. Garg and R. B. Haynes	2013	Features of effective computerised clinical decision support systems: Meta-regression of 162 randomised trials
534	G. Rouleau, M. P. Gagnon, J. Côté, J. Payne-Gagnon, E. Hudson and C. A. Dubois	2017	Impact of information and communication technologies on nursing care: Results of an overview of systematic reviews
535	R. Rozenblum, J. Donzé, P. M. Hockey, E. Guzdar, M. A. Labuzetta, E. Zimlichman and D. W. Bates	2013	The impact of medical informatics on patient satisfaction: A USA-based literature review
536	A. Rudolph, J. Vaughn, N. Crego, R. Hueckel, M. Kuszajewski, M. Molloy, R. Brisson Iii and R. J. Shaw	2017	Integrating Telepresence Robots Into Nursing Simulation
537	S. Rus, T. Grosse-Puppendahl and A. Kuijper	2014	Recognition of bed postures using mutual capacitance sensing
538	P. W. Rushton, B. W. Mortenson, P. Viswanathan, R. H. Wang, W. C. Miller and L. Hurd Clarke	2017	Intelligent power wheelchair use in long-term care: potential users' experiences and perceptions
539	T. Ryall, B. K. Judd and C. J. Gordon	2016	Simulation-based assessments in health professional education: A systematic review
540	J. Ryan, B. Doster, S. Daily, R. Ryan and C. Lewis	2015	Perioperative workflow and patient care documentation perpetuated through electronic medical records via integrated hospital information systems

541	S. Šabanović, C. C. Bennett, W. L. Chang and L. Huber	2013	PARO robot affects diverse interaction modalities in group sensory therapy for older adults with dementia
542	A. M. Sabelli, T. Kanda and N. Hagita	2011	A Conversational Robot in an Elderly Care Center: An Ethnographic Study
543	F. Sadoughi, K. Kimiafar, M. Ahmadi and M. T. Shakeri	2013	Determining of factors influencing the success and failure of hospital information system and their evaluation methods: A systematic review
544	O. Sahota, A. Drummond, D. Kendrick, M. J. Grainge, C. Vass, T. Sach, J. Gladman and M. Avis	2014	REFINE (REducing Falls in In-patieNt Elderly) using bed and bedside chair pressure sensors linked to radio-pagers in acute hospital care: a randomised controlled trial
545	A. Salekin, H. Wang, K. Williams and J. Stankovic	2017	DAVE: Detecting Agitated Vocal Events
546	J. Salinas, K. K. Chung, E. A. Mann, L. C. Cancio, G. C. Kramer, M. L. Serio-Melvin, E. M. Renz, C. E. Wade and S. E. Wolf	2011	Computerized decision support system improves fluid resuscitation following severe burns: An original study
547	V. L. Salyers, L. Carter, C. Antoniazzi and S. Johnson	2013	Evaluating the Effectiveness of a Clinical Tracking System for Undergraduate Nursing Students
548	C. Sanders, A. Rogers, R. Bowen, P. Bower, S. Hirani, M. Cartwright, R. Fitzpatrick, M. Knapp, J. Barlow, J. Hendy, T. Chrysanthaki, M. Bardsley and S. Newman	2012	Exploring barriers to participation and adoption of telehealth and telecare within the Whole System Demonstrator trial: a qualitative study
549	W. Sansrimahachai and M. Toahchoodee	2016	Mobile-phone based immobility tracking system for elderly care
550	J. Saunders, D. S. Syrdal, K. L. Koay, N. Burke and K. Dautenhahn	2016	“Teach Me–Show Me”—End-User Personalization of a Smart Home and Companion Robot
551	M. C. Schall, L. Cullen, P. Pennathur, H. Chen, K. Burrell and G. Matthews	2017	Usability Evaluation and Implementation of a Health Information Technology Dashboard of Evidence-Based Quality Indicators
552	L. Schiatti, J. Tessadori, G. Barresi, L. S. Mattos and A. Ajoudani	2017	Soft brain-machine interfaces for assistive robotics: A novel control approach
553	T. Schmidt, A. Lassen and U. K. Wiil	2016	A Patient Deterioration Warning System for Boosting Situational Awareness of Monitored Patients
554	R. Schnall	2015	Short message service use in clinical care through a simulation activity
555	R. R. Schoville	2017	Discovery of Implementation Factors That Lead to Technology Adoption in Long-Term Care

556	G. Schreier, M. Schwarz, R. Modre-Osprian, P. Kastner, D. Scherr and F. Fruhwald	2013	Design and evaluation of a multimodal mHealth based medication management system for patient self administration
557	J. Schuld, T. Schäfer, S. Nickel, P. Jacob, M. K. Schilling and S. Richter	2011	Impact of IT-supported clinical pathways on medical staff satisfaction. A prospective longitudinal cohort study
558	J. L. Scott, S. Dawkins, M. G. Quinn, K. Sanderson, K. E. Elliott, C. Stirling, B. Schuz and A. Robinson	2016	Caring for the carer: a systematic review of pure technology-based cognitive behavioral therapy (TB-CBT) interventions for dementia carers
559	Y. S. Sefidgar, K. E. MacLean, S. Yohanan, H. F. M. Van Der Loos, E. A. Croft and E. J. Garland	2016	Design and Evaluation of a Touch-Centered Calming Interaction with a Social Robot
560	H. H. Seibert, R. R. Maddox, E. A. Flynn and C. K. Williams	2014	Effect of barcode technology with electronic medication administration record on medication accuracy rates
561	J. K. Sheba, R. E. Mohan and E. A. Martínez García	2012	Easiness of acceptance metric for effective human robot interactions in therapeutic pet robots
562	A. W. Shee, B. Phillips, K. Hill and K. Dodd	2014	Feasibility, acceptability, and effectiveness of an electronic sensor bed/chair alarm in reducing falls in patients with cognitive impairment in a subacute ward
563	Z. Shen and Y. Wu	2016	Investigation of Practical Use of Humanoid Robots in Elderly Care Centres
564	H. Shin, S. Sok, K. S. Hyun and M. J. Kim	2015	Competency and an active learning program in undergraduate nursing education
565	M. A. Shinnick, M. A. Woo and J. C. Mentes	2011	Human Patient Simulation: State of the Science in Prelicensure Nursing Education
566	A. A. N. Shirehjini, A. Yassine and S. Shirmohammadi	2012	Equipment location in hospitals using RFID-based positioning system
567	J. T. Shuffitt	2011	Utilization and influence of health information technology on Kentucky advanced practice registered nurses' clinical decision making
568	J. Shukla, M. Barreda-Ángeles, J. Oliver and D. Puig	2017	Effectiveness of socially assistive robotics during cognitive stimulation interventions: Impact on caregivers
569	K. K. Shyu, Y. J. Chiu, P. L. Lee, M. H. Lee, J. J. Sie, C. H. Wu, Y. T. Wu and P. C. Tung	2013	Total Design of an FPGA-Based Brain–Computer Interface Control Hospital Bed Nursing System
570	S. R. Simon, C. A. Keohane, M. Amato, M. Coffey, B. Cadet, E. Zimlichman and D. W. Bates	2013	Lessons learned from implementation of computerized provider order entry in 5 community hospitals: A qualitative study
571	P. M. Sinclair, A. Kable, T. Levett-Jones and D. Booth	2016	The effectiveness of Internet-based e-learning on clinician behaviour and patient outcomes: A systematic review
572	M. M. Soares, K. Jacobs, Y.-C. Chen and C.-Y. Leung	2012	Exploring functions of the lost seeking devices for people with dementia

573	P. S. Sockolow, K. H. Bowles, H. P. Lehmann, P. A. Abbott and J. P. Weiner	2012	Community-based, interdisciplinary geriatric care team satisfaction with an electronic health record: a multimethod study
574	L. Song	2013	Evaluating the Relationship between Patient Safety Culture and the Behavioral Intention to Use Bar Code Medication Administration among Registered Nurses in Hospitals
575	V. D. Souza-Junior, I. A. C. Mendes, A. Mazzo and S. Godoy	2016	Application of telenursing in nursing practice: an integrative literature review
576	V. D. d. Souza-Junior, I. A. C. Mendes, A. Mazzo, S. d. Godoy and C. A. d. Santos	2017	Telenursing Intervention for Clean Intermittent Urinary Catheterization Patients: A Pilot Study
577	A. K. Sowan and L. S. Jenkins	2013	Designing, delivering and evaluating a distance learning nursing course responsive to students needs
578	F. Stertz, J. Mangler and S. Rinderle-Ma	2017	NFC-based task enactment for automatic documentation of treatment processes
579	K. Steurbaut, K. Colpaert, S. Van Hoecke, S. Steurbaut, C. Danneels, J. Decruyenaere and F. De Turck	2012	Design and evaluation of a service oriented architecture for paperless ICU tarification
580	A. Steventon, M. Bardsley, J. Billings, J. Dixon, H. Doll, M. Beynon, S. Hirani, M. Cartwright, L. Rixon, M. Knapp, C. Henderson, A. Rogers, J. Hendy, R. Fitzpatrick and S. Newman	2013	Effect of telecare on use of health and social care services: findings from the Whole Systems Demonstrator cluster randomised trial
581	I. Strand, L. Gulbrandsen, Å. Slettebø and D. Nåden	2017	Digital recording as a teaching and learning method in the skills laboratory
582	K. Strickland, C. Gray and G. Hill	2012	The use of podcasts to enhance research-teaching linkages in undergraduate nursing students
583	J. L. Styron	2013	The impact of technology attitudes and skills of rural health clinic nurses on the level of adoption of electronic health records in Mississippi
584	P. Subramaniam and B. Woods	2016	Digital life storybooks for people with dementia living in care homes: an evaluation
585	C. Suebsin and N. Gerdsri	2011	Lessons learned from IT adoption in healthcare organizations: A comparative study
586	M. R. Summerfield, F. J. Seagull, N. Vaidya and Y. Xiao	2011	Use of pharmacy delivery robots in intensive care units
587	C. Sunnqvist, K. Karlsson, L. Lindell and U. Fors	2016	Virtual patient simulation in psychiatric care – A pilot study of digital support for collaborate learning
588	R. Suzuki and N. Kobayashi	2014	Development of assistive devices for feeding gastrostomy

589	S. Suzuki, T. Yokoishi, H. Hada, J. Mitsugi, O. Nakamura and J. Murai	2011	Bidirectional medication support system for medical staff and home care patients
590	A. Takian, A. Sheikh and N. Barber	2012	We are bitter, but we are better off: Case study of the implementation of an electronic health record system into a mental health hospital in England
591	A. Talaei-Khoei, L. Lewis, T. T. Khoei, A. H. Ghapanchi and S. Vichitvanichphong	2015	Seniors' perspective on perceived transfer effects of assistive robots in elderly care: Capability approach analysis
592	L. T. Tam, A. C. Valera, H.-P. Tan and C. Koh	2016	Online Detection of Behavioral Change Using Unobtrusive Eldercare Monitoring System
593	P. Tangtisanon	2016	Healthcare system for elders with automatic drug label detection
594	L. Tapper, H. Quinn, J. Kerry and K. G. Brown	2012	Introducing Handheld Computers into Home Care
595	A. Tariq, J. Westbrook, M. Byrne, M. Robinson and M. T. Baysari	2017	Applying a human factors approach to improve usability of a decision support system in tele-nursing
596	J. Taylor, E. Coates, L. Brewster, G. Mountain, B. Wessels and M. S. Hawley	2015	Examining the use of telehealth in community nursing: identifying the factors affecting frontline staff acceptance and telehealth adoption
597	S. Taylor, M. J. Allsop, H. L. Bekker, M. I. Bennett and B. M. Bewick	2017	Identifying professionals' needs in integrating electronic pain monitoring in community palliative care services: An interview study
598	A. E. Tchalla, F. Lachal, N. Cardinaud, I. Saulnier, V. Rialle and P.-M. Preux	2013	Preventing and managing indoor falls with home-based technologies in mild and moderate Alzheimer's disease patients: pilot study in a community dwelling
599	H. E. Thomassen and B. A. Farshchian	2016	A technology-enhanced service for person-centered dementia care: Preliminary results from a field trial
600	D. Thompson, K. R. Fisher and R. Kayess	2012	The Role of Assistive Technology in Supporting People with Disabilities and Complex Care Needs: A Literature Review
601	G. Thompson, J. C. O'Horo, B. W. Pickering and V. Herasevich	2015	Impact of the Electronic Medical Record on Mortality, Length of Stay, and Cost in the Hospital and ICU: A Systematic Review and Metaanalysis
602	T. L. Thompson	2011	Meaningful use of simulation as an educational method in nursing programs
603	L. Tiberio, A. Cesta, G. Cortellessa, L. Padua and A. R. Pellegrino	2012	Assessing affective response of older users to a telepresence robot using a combination of psychophysiological measures

604	B. R. Tielbur, D. E. Rice Cella, A. Currie, J. D. Roach, B. Mattingly, J. Boone, C. Watwood, A. McGauran, H. S. Kirshner and P. D. Charles	2015	Discharge huddle outfitted with mobile technology improves efficiency of transitioning stroke patients into follow-up care
605	J. J. Tieman, K. Swetenham, D. D. Morgan, T. H. To and D. C. Currow	2016	Using telehealth to support end of life care in the community: a feasibility study
606	L. Tieu, U. Sarkar, D. Schillinger, J. D. Ralston, N. Ratanawongsa, R. Pasick and C. R. Lyles	2015	Barriers and facilitators to online portal use among patients and caregivers in a safety net health care system: A qualitative study
607	J. S. L. Ting, A. H. C. Tsang, A. W. H. Ip and G. T. S. Ho	2011	RF-Medisys: a radio frequency identification-based electronic medical record system for improving medical information accessibility and services at point of care
608	K. L. H. Ting, D. Voilmy, A. Iglesias, J. C. Pulido, J. García, A. Romero-Garcés, J. P. Bandera, R. Marfil and D. Á	2017	Integrating the users in the design of a robot for making Comprehensive Geriatric Assessments (CGA) to elderly people in care centers
609	P. Tiwari, J. Warren, K. Day, B. MacDonald, C. Jayawardena, I. H. Kuo, A. Igic and C. Datta	2011	Feasibility Study of a Robotic Medication Assistant for the Elderly
610	S. Torp, P. C. Bing-Jonsson and E. Hanson	2013	Experiences with using information and communication technology to build a multi-municipal support network for informal carers
611	M. Tower, S. Latimer and J. Hewitt	2014	Social networking as a learning tool: Nursing students' perception of efficacy
612	M. Trent, C. Gaydos, J. Perin, S. E. Chung, S. Huettner, J. Anders, R. Rothman and A. Butz	2017	Effectiveness of technology enhanced community health nursing (TECH-N) for adolescents and young adult women with pelvic inflammatory disease
613	C. Tsiourti, E. Joly, C. Wings, M. B. Moussa and K. Wac	2014	Virtual Assistive Companions for Older Adults: Qualitative Field Study and Design Implications
614	A. Tsuji, T. Yonezawa, H. Yamazoe, S. Abe, N. Kuwahara and K. Morimoto	2012	Proposal and evaluation of the toilet timing suggestion method for the elderly
615	N. Vadiei, C. Shuman, M. Murthy and M. Daley	2017	Optimization of intelligent infusion pump technology to minimize vasopressor pump programming errors
616	P. C. Vadillo and E. S. Rojo	2016	Maximizing Healthcare Professionals' Use of New Computer Technologies in a Small, Urban Hospital's Critical Care Unit

617	S. M. Valenti, L. Aguera-Ortiz, R. J. Olazaran, R. C. Mendoza, M. A. Perez, P. I. Rodriguez, R. E. Osa, S. A. Barrios, C. V. Herrero, C. L. Carrasco, R. S. Felipe, A. J. Lopez, S. B. Leon, P. J. Canas, R. F. Martin and M. P. Martinez	2015	Social robots in advanced dementia
618	T. Valerie, K. L. Choy, P. K. Y. Siu, H. Y. Lam, G. T. S. Ho and S. W. Y. Cheng	2016	An intelligent performance assessment system for enhancing the service quality of home care nursing staff in the healthcare industry
619	J.-W. van ?t Klooster, C. Combes and B.-J. van Beijnum	2012	Towards Decision Support for a Home Care Services Platform
620	L. A. van der Heide, C. G. Willems, M. D. Spreeuwenberg, J. Rietman and L. P. de Witte	2012	Implementation of CareTV in care for the elderly: The effects on feelings of loneliness and safety and future challenges
621	M. van der Lende, F. M. E. Cox, G. H. Visser, J. W. Sander and R. D. Thijs	2016	Value of video monitoring for nocturnal seizure detection in a residential setting
622	H. G. Van der Roest, J. Wenborn, C. Pastink, R. M. Droes and M. Orrell	2017	Assistive technology for memory support in dementia
623	A. E. Vandenberg, B.-J. van Beijnum, V. G. P. Overdevest, E. Capezuti and T. M. Iijohnson	2017	US and Dutch nurse experiences with fall prevention technology within nursing home environment and workflow: A qualitative study
624	A. Vankipuram, P. Khanal, A. Ashby, M. Vankipuram, A. Gupta, D. DrummGurnee, K. Josey and M. Smith	2014	Design and Development of a Virtual Reality Simulator for Advanced Cardiac Life Support Training
625	D. Vanneste, B. Vermeulen and A. Declercq	2013	Healthcare professionals' acceptance of BelRAI, a web-based system enabling person-centred recording and data sharing across care settings with interRAI instruments: A UTAUT analysis
626	F. Vannieuwenborg, F. Ongenae, P. Demyttenaere, L. V. Poucke, J. V. Ooteghem, S. Verstichel, S. Verbrugge, D. Colle, F. D. Turck and M. Pickavet	2014	Techno-economic evaluation of an ontology-based nurse call system via discrete event simulations
627	U. Varshney	2011	Wireless Medication Management System: Design and performance evaluation
628	I. Vedel, S. Akhlaghpour, I. Vaghefi, H. Bergman and L. Lapointe	2013	Health information technologies in geriatrics and gerontology: a mixed systematic review
629	M. Verkuyl, L. Atack, P. Mastrilli and D. Romaniuk	2016	Virtual gaming to develop students' pediatric nursing skills: A usability test

630	S. Vichitvanichphong, D. Kerr, A. Talaei-Khoei and A. H. Ghapanchi	2013	Analysis of research in adoption of assistive technologies for aged care
631	S. Vichitvanichphong, A. Talaei-Khoei, D. Kerr and A. H. Ghapanchi	2014	Adoption of Assistive Technologies for Aged Care: A Realist Review of Recent Studies
632	M. Vincze, W. Zagler, L. Lammer, A. Weiss, A. Huber, D. Fischinger, T. Koertner, A. Schmid and C. Gisinger	2014	Towards a Robot for Supporting Older People to Stay Longer Independent at Home
633	A. Voit, D. Weber, E. Stowell and N. Henze	2017	Caloo: An Ambient Pervasive Smart Calendar to Support Aging in Place
634	K. Vowden and P. Vowden	2013	A pilot study on the potential of remote support to enhance wound care for nursing-home patients
635	K. Wada, Y. Takasawa and T. Shibata	2014	Robot therapy at facilities for the elderly in Kanagawa prefecture - a report on the experimental result of the first month
636	E. Wagemaker, T. J. Dekkers, J. A. Agelink van Rentergem, K. M. Volkers and H. M. Huizenga	2017	Advances in Mental Health Care: Five N = 1 Studies on the Effects of the Robot Seal Paro in Adults With Severe Intellectual Disabilities
637	B. J. Wakefield and M. Vaughan-Sarrazin	2017	Home Telehealth and Caregiving Appraisal in Chronic Illness
638	G. S. Walia, A. L. Wong, A. Y. Lo, G. A. Mackert, H. M. Carl, R. A. Pedreira, R. Bello, C. S. Aquino, W. V. Padula and J. M. Sacks	2016	Efficacy of Monitoring Devices in Support of Prevention of Pressure Injuries: Systematic Review and Meta-analysis
639	K. Walsh and A. Callan	2011	Perceptions, Preferences, and Acceptance of information and communication technologies in older-adult community care settings in Ireland: A case-study and ranked-care program analysis
640	H. Wang, G. G. Grindle, J. Candiotti, C. Chung, M. Shino, E. Houston and R. A. Cooper	2012	The Personal Mobility and Manipulation Appliance (PerMMA): A robotic wheelchair with advanced mobility and manipulation
641	H. Wang, J. Xu, G. Grindle, J. Vazquez, B. Salatin, A. Kelleher, D. Ding, D. M. Collins and R. A. Cooper	2013	Performance evaluation of the personal mobility and manipulation appliance (PerMMA)
642	R. H. Wang, S. M. Gorski, P. J. Holliday and G. R. Fernie	2011	Evaluation of a Contact Sensor Skirt for an Anti-Collision Power Wheelchair for Older Adult Nursing Home Residents With Dementia: Safety and Mobility
643	R. H. Wang, P. C. Kontos, P. J. Holliday and G. R. Fernie	2011	The experiences of using an anti-collision power wheelchair for three long-term care home residents with mild cognitive impairment
644	R. H. L. Wang	2011	Enabling Power Wheelchair Mobility with Long-Term Care Home Residents with Cognitive Impairments

645	T. Wang, Y. Wang and J. Moczygemba	2014	Organizational factors influencing health information technology adoption in long-term-care facilities
646	L. Wanner, E. André, J. Blat, S. Dasiopoulou, M. Farrús, T. Fraga, E. Kamateri, F. Lingenfelser, G. Llorach, O. Martínez, G. Meditskos, S. Mille, W. Minker, L. Pragst, D. Schiller, A. Stam, L. Stellingwerff, F. Sukno, B. Vieru and S. Vrochidis	2017	Design of a Knowledge-Based Agent as a Social Companion
647	A. Weakley, J. W. Tam, C. Van Son and M. Schmitter-Edgecombe	2017	Effectiveness of a video-based aging services technology education program for health care professionals
648	L. Webb, J. Clough, D. O'Reilly, D. Wilmott and G. Witham	2017	The utility and impact of information communication technology (ICT) for pre-registration nurse education: A narrative synthesis systematic review
649	D. Webster and O. Celik	2014	Systematic review of Kinect applications in elderly care and stroke rehabilitation
650	G. Webster and V. L. Hanson	2014	Technology for Supporting Care Staff in Residential Homes
651	C. Weiß	2013	Unterstützung Pflegebedürftiger durch technische Assistenzsysteme (Abschlussbericht)
652	L. J. Wekre, L. Melby and A. Grimsmo	2011	Early experiences with the multidose drug dispensing system -- A matter of trust?
653	C. White, S. McIlfatrick, L. Dunwoody and M. Watson	2015	Supporting and improving community health services-a prospective evaluation of ECHO technology in community palliative care nursing teams
654	J. C. Whitehead, S. A. Gambino, J. D. Richter and J. D. Ryan	2015	Focus group reflections on the current and future state of cognitive assessment tools in geriatric health care
655	A. Wickramasinghe, D. C. Ranasinghe, C. Fumeaux, K. D. Hill and R. Visvanathan	2017	Sequence Learning with Passive RFID Sensors for Real-Time Bed-Egress Recognition in Older People
656	A. Wickramasinghe, R. L. Shinmoto Torres and D. C. Ranasinghe	2017	Recognition of falls using dense sensing in an ambient assisted living environment
657	M. Wieck, B. Blake, C. Sellick, D. Kenron, D. DeVries, S. Terry and S. Krishnaswami	2017	Utilizing technology to improve intraoperative family communication
658	J. J. Willemse and V. Bozalek	2015	Exploration of the affordances of mobile devices in integrating theory and clinical practice in an undergraduate nursing programme
659	W. Williams	2014	Factors that Affect Bar Code Medication Administration Technology Acceptance

660	B. Williamson, T. Aplin, D. de Jonge and M. Goyne	2017	Tracking down a solution: exploring the acceptability and value of wearable GPS devices for older persons, individuals with a disability and their support persons
661	S. Winslow, S. Jackson, K. Blakeney, L. Cook, J. W. Reed, K. Zimbro and C. Parker	2016	Multisite assessment of nursing continuing education learning needs using an electronic tool
662	J. T. Wiseman, S. Fernandes-Taylor, M. L. Barnes, A. Tomsejova, R. S. Saunders and K. C. Kent	2015	Conceptualizing smartphone use in outpatient wound assessment: Patients' and caregivers' willingness to use technology
663	R. A. Wittmann-Price, L. D. Kennedy and C. Godwin	2012	Use of personal phones by senior nursing students to access health care information during clinical education: Staff Nurses' and students' perceptions
664	K. H. Wolf, K. Hetzer, H. M. zu Schwabedissen, B. Wiese and M. Marschollek	2013	Development and pilot study of a bed-exit alarm based on a body-worn accelerometer
665	A. Wong Shee, B. Phillips, K. Hill and K. Dodd	2014	Feasibility, Acceptability, and Effectiveness of an Electronic Sensor Bed/Chair Alarm in Reducing Falls in Patients With Cognitive Impairment a Subacute Ward
666	J. Woo, K. Wada and N. Kubota	2012	Robot Partner System for elderly people care by using sensor network
667	J. L. Wood and J. S. Burnette	2012	Enhancing patient safety with intelligent intravenous infusion devices: Experience in a specialty cardiac hospital
668	K. Words	2014	A Web-Based Intelligent Tutoring System Teaching Health Care Technology
669	R. Wu, P. Rossos, S. Quan, S. Reeves, V. Lo, B. Wong, M. Cheung and D. Morra	2011	An evaluation of the use of smartphones to communicate between clinicians: A mixed-methods study
670	T. T. Wu, S. H. Huang, M. Y. Chung and Y. M. Huang	2013	Group Investigation Learning with Google Plus for Public Health Nursing Practice Course
671	Y.-H. Wu, M. Chetouani, V. Cristancho-Lacroix, J. L. Maître, C. Jost, B. L. Pevedic, D. Duhaut, C. Granata and A.-S. Rigaud	2011	ROBADOM: The Impact of a Domestic Robot on Psychological and Cognitive State of the Elderly with Mild Cognitive Impairment
672	D. Xie, Y. Lin, R. Grupen and A. Hanson	2011	Intention-based coordination and interface design for human-robot cooperative search
673	P. H. Yager, M. Clark, B. M. Cummings and N. Noviski	2017	Parent Participation in Pediatric Intensive Care Unit Rounds via Telemedicine: Feasibility and Impact
674	M. Yamamoto, N. Takabayashi, K. Ono, T. Watanabe and Y. Ishii	2014	Development of a nursing communication education support system using nurse-patient embodied avatars with a smile and eyeball movement model

675	N. Yamashita, H. Kuzuoka, K. Hirata, T. Kudo, E. Aramaki and K. Hattori	2017	Changing Moods: How Manual Tracking by Family Caregivers Improves Caring and Family Communication
676	H. J. Yazici	2014	An exploratory analysis of hospital perspectives on real time information requirements and perceived benefits of RFID technology for future adoption
677	B. Yeaman, K. J. Ko and R. A. d. Castillo	2015	Care Transitions in Long-term Care and Acute Care: Health Information Exchange and Readmission Rates
678	C. Yen-Ting, H. Chun-Ju, Z. Jia-Hong, H. Min-Wei and Z. Jia-Ying	2016	The development of image tracing technology in the surveillance system for elder's living in home
679	C. Yi-Sheng, L. Hsin-Ju and L. Yuan-Hsiang	2014	Using wireless measuring devices and Tablet PC to improve the efficiency of vital signs data collection in hospital
680	Y. Yixiao, C. Innocenti, G. Nero, H. L. én and G. Irene Yu-Hua	2015	Fall detection in RGB-D videos for elderly care
681	L. B. Young, P. S. Chan, P. Cram, L. B. Young, P. S. Chan and P. Cram	2011	Staff acceptance of tele-ICU coverage: a systematic review
682	M. Yu, Y. Yu, A. Rhuma, S. M. R. Naqvi, L. Wang and J. A. Chambers	2013	An Online One Class Support Vector Machine-Based Person-Specific Fall Detection System for Monitoring an Elderly Individual in a Room Environment
683	M. J. Yuan, G. M. Finley, J. Long, C. Mills and R. K. Johnson	2013	Evaluation of user interface and workflow design of a bedside nursing clinical decision support system
684	Y. Yun and I. Y. H. Gu	2015	Human fall detection via shape analysis on Riemannian manifolds with applications to elderly care
685	S. Yusif, J. Soar and A. Hafeez-Baig	2016	Older people, assistive technologies, and the barriers to adoption: A systematic review
686	M. M. Yusof	2015	A case study evaluation of a Critical Care Information System adoption using the socio-technical and fit approach
687	C. Zaccarelli, G. Cirillo, S. Passuti, R. Annicchiarico and F. Barban	2013	Computer-based cognitive intervention for dementia Sociable: motivating platform for elderly networking, mental reinforcement and social interaction
688	P. E. Zadeh	2015	Adoption and implementation of Health Information Exchange (HIE): An interpretative review
689	M. Zahabi, D. B. Kaber and M. Swangnetr	2015	Usability and Safety in Electronic Medical Records Interface Design: A Review of Recent Literature and Guideline Formulation
690	M. Zaidan, F. Rustom, N. Kassem, S. Al Yafei, L. Peters and M. I. M. Ibrahim	2016	Nurses' perceptions of and satisfaction with the use of automated dispensing cabinets at the Heart and Cancer Centers in Qatar: a cross-sectional study

691	A. Zaman and M. Bhuiyan	2014	Usabilty evaluation of the MumIES (Multimodal Interface based Education and Support) system for the children with special needs in Bangladesh
692	R. A. Zapatán, E. E. Armijos, L. Serpa-Andrade and E. Pinos	2017	Analysis of a nurse call system implementation using a wireless sensors network
693	N. Zayim and D. Ozel	2015	Factors affecting nursing students' readiness and perceptions toward the use of mobile technologies for learning
694	M. Zhang, K. Bingham, K. Kantarovich, J. Laidlaw, D. Urbach, S. Sockalingam and R. Ho	2016	Inter-professional delirium education and care: a qualitative feasibility study of implementing a delirium Smartphone application
695	N. Zhang, S. F. Lu, B. Xu, B. Wu, R. Rodriguez-Monguio and J. Gurwitz	2016	Health Information Technologies: Which Nursing Homes Adopted Them?
696	Y. Zhang, P. Yu and J. Shen	2012	The benefits of introducing electronic health records in residential aged care facilities: A multiple case study
697	W. Zhao, Q. Wu, D. D. Espy, M. A. Reinthal, X. Luo and Y. Peng	2017	A feasibility study on using a Kinect-based human motion tracking system to promote safe patient handling
698	W. Zhao, X. Zhou, H. Ni and Q. Lin	2014	A light-weight system for detecting indoor wandering of demented elders living alone
699	X. Zhao, A. M. Naguib and S. Lee	2014	Octree Segmentation Based Calling Gesture Recognition for Elderly Care Robot
700	J. Zhou, D. B. Liu, J. W. Zhong, Z. Y. Huang, S. Y. Qiu, Y. P. Zhou and X. H. Yi	2012	Feasibility of a remote monitoring system for home-based non-invasive positive pressure ventilation of children and infants
701	X. Zhou, X. Zhu, M. Huang and W. Chen	2013	Automatic monitoring of sleep behaviour in nursing home residents
702	C. Zhu, W. Sheng and M. Liu	2015	Wearable Sensor-Based Behavioral Anomaly Detection in Smart Assisted Living Systems
703	X. Zhu, X. Zhou, W. Chen, K. I. Kitamura and T. Nemoto	2014	Estimation of Sleep Quality of Residents in Nursing Homes Using an Internet-Based Automatic Monitoring System
704	J. Zhuang, R. Fang, X. Feng, X. Xu, L. Liu, Q. Bai, H. Tang, Z. Zhao and S. Chen	2013	The impact of human-computer interaction-based comprehensive training on the cognitive functions of cognitive impairment elderly individuals in a nursing home
705	M. Ziefle, C. Rocker and A. Holzinger	2011	Medical Technology in Smart Homes: Exploring the User's Perspective on Privacy, Intimacy and Trust
706	E. Zimlichman, J. Terrence, D. Argaman, Z. Shinar and H. Brown	2012	Effect of contactless continuous patient monitoring in a medical-surgical unit on intensive care unit transfers: a controlled clinical trial

707	C. Zimmermann, J. Zeilfelder, T. Bloecher, M. Diehl, S. Essig and W. Stork	2017	Evaluation of a smart drink monitoring device
708	A. Rocca, J. M. Pignat, L. Berney, J. Johr, D. Van de Ville, R. T. Daniel, M. Levivier, L. Hirt, A. R. Luft, E. Grouzmann and K. Diserens	2016	Sympathetic activity and early mobilization in patients in intensive and intermediate care with severe brain injuries: a preliminary prospective randomized study
709	P. Ewig	2016	Projekt Aaladin : Anwendung von akustischen und lautbasierten Erkennertechnologien zur Unterstützung pflegender Dienstleister : Schlussbericht : Laufzeit des Vorhabens: 01. Oktober 2012 - 31. Dezember 2015
710	S. C. Trukeschitz B., Ring-Dimitriou S.	2018	Smartes Betreutes Wohnen: Nutzung, Systemakzeptanz und Wirkungen von „meinZentrAAL“
711	N. Loepthien, T. Jehnichen, J. Hauser, B. Schullcke and K. Möller	2016	Development of a low-cost senor based aid for visually impaired people
712	B. Weber-Fiori, A. Rölle and M. Winter	2017	Geruchssensorik in der professionellen pflegerischen Langzeitversorgung (GeppV) – Chancen und Risiken für die Handlungspraxis Pflegender
713	S. Müller and A. Hein	2016	Multi-Target Data Association in Binary Sensor Networks for Ambulant Care Support
714	T. Frenken, R. Eckert, A. Jüptner and A. Hein	2015	AmbiAct - RRI Industry case study
715	S. Teipel, C. Heine, A. Hein, F. Krüger, A. Kutschke, S. Kernebeck, M. Halek, S. Bader and T. Kirste	2017	Multidimensional assessment of challenging behaviors in advanced stages of dementia in nursing homes—The insideDEM framework
